# Moisture Dynamics of Wood-Based Panels and Wood Fibre Insulation Materials

**DOI:** 10.3389/fpls.2022.951175

**Published:** 2022-07-14

**Authors:** Liselotte De Ligne, Joris Van Acker, Jan M. Baetens, Salah Omar, Bernard De Baets, Lisbeth G. Thygesen, Jan Van den Bulcke, Emil E. Thybring

**Affiliations:** ^1^Laboratory of Wood Technology (UGent-Woodlab), Department of Environment, Ghent University (UGent), Ghent, Belgium; ^2^Research Unit Knowledge-Based Systems (KERMIT), Department of Data Analysis and Mathematical Modelling, Ghent University (UGent), Ghent, Belgium; ^3^Centre for X-ray Tomography (UGCT), Ghent University, Gent, Belgium; ^4^Bioresource Chemistry and Technology, Department of Geosciences and Natural Resource Management, University of Copenhagen, Copenhagen, Denmark

**Keywords:** moisture dynamics, wood-based panels, wood fibre insulation, service life, X-ray CT, LFNMR, ATR-FTIR

## Abstract

Moisture performance is an important factor determining the resistance of wood-based building materials against fungal decay. Understanding how material porosity and chemistry affect moisture performance is necessary for their efficient use, as well as for product optimisation. In this study, three complementary techniques (X-ray computed tomography, infrared and low-field NMR spectroscopy) are applied to elucidate the influence of additives, manufacturing process and material structure on the liquid water absorption and desorption behaviour of a selection of wood-based panels, thermally modified wood and wood fibre insulation materials. Hydrophobic properties achieved by thermal treatment or hydrophobic additives such as paraffin and bitumen, had a major influence on water absorption and desorption rates. When hydrophobic additives did not play a role, pore distributions and manufacturing process had a decisive influence on the amount and rate of absorption and desorption. In that case, a higher porosity resulted in a higher water absorption rate. Our results show that there is a clear potential for tailoring materials towards specific moisture performance by better understanding the influence of different material characteristics. This is useful both for achieving desired moisture buffering as well as to increase service life of wood-based materials. From a sustainability perspective, fit-for-purpose moisture performance is often easier to achieve and preferred than wood protection by biocide preservative treatments.

## Introduction

Wood is a renewable resource and wood-based building materials are produced with considerably less energy and associated carbon emissions than many traditional building materials (Sathre and O’Connor, 2010; Amiri et al., 2020; Churkina et al., 2020). This makes them essential building elements in the much-needed transition towards a more sustainable building industry (EC, 2022a,b). Wood-based building materials are hygroscopic, meaning that they can attract, hold and release water molecules (Skaar, 1988). Due to their hygroscopic nature, wood and other bio-based building materials can act as moisture-buffering materials (Rode et al., 2005; Li et al., 2012). This is an excellent characteristic, as a more constant air humidity reduces the energy needed for heating and cooling of an interior space and improves the air quality (Osanyintola and Simonson, 2006; Lozhechnikova et al., 2015). In the hygroscopic moisture range, i.e., from 0 to about 95–98% relative humidity (RH), the moisture is absorbed by the wood cell walls and bound to the cell wall polymers through hydrogen bonding (Fredriksson, 2019). When liquid water is present, for instance due to condensation or leakage indoors or in outdoor exposure applications, hygroscopic materials take up water in cell lumen, pits and macro voids (such as the space between strands in oriented strand board) and the risk of fungal decay increases (CEN EN 335 standard, 2013; Brischke and Alfredsen, 2020). Consequently, the interaction with moisture has an important impact on the performance and service life of wood-based building materials, both on fungal resistance (Ross, 2010; Jones and Brischke, 2017) and on mechanical properties such as strength and stiffness (Gibson, 1965; Gerhards, 1982; CEN EN 1995-1-1 standard, 2004). The service life of a material is the period of time after installation during which the product needs to meet its minimal performance requirements and no replacement is needed (Kutnik et al., 2020). This depends on the natural durability of the material and the environmental conditions (Brischke and Rapp, 2010).

The European Committee of standardization has defined five ‘use classes’ (UC) for applications of wood, with corresponding harmful organisms that can occur in those application types (CEN EN 335 standard, 2013). The risk of fungal decay is strongly related to the presence of moisture: interior and dry (UC1), interior or under roof, not exposed to the weather, but with the possibility of condensation (UC2), exterior without soil contact, but exposed to the weather (UC3) which includes two sub-classes [limited moist conditions (3.1) and persistently moist conditions (3.2)], and finally, exterior in contact with soil or freshwater (UC4). Ideally, materials with a low natural durability are applied in applications with low risk (UC1), while materials with a high intrinsic durability or an enhanced durability are applied in applications with a high risk (UC3 and 4; fit-for-purpose). Note that similar hazard classifications exist in Australia, New-Zealand, and the United States (AS, 2005; AWPA, 2022). Additionally, Eurocode 5 (CEN EN 1995-1-1 standard, 2004) makes use of three ‘service classes’ to predict strength and deformation of construction timber under defined environmental conditions, distinguishing between environmental conditions where the relative humidity exceeds 65% (SC 1) and 85% (SC 2) only a few weeks per year, and the corresponding equilibrium moisture content of softwood timber is expected to remain below 12 and 20% moisture content, respectively. The third service class (SC 3) is characterized by ‘climatic conditions leading to higher moisture contents than in SC 2’.

When it comes to applications in use classes with a fungal decay risk, usually wood products with a high natural durability or wood treated with biocide preservatives (fungicidal components) are recommended, although standard EN 460 also states that wood species with a low water permeability will acquire lower moisture contents in intermittent wetting conditions and will consequently have a reduced risk in UC2 and UC3 (CEN EN 460, 1994; CEN prEN 460, 2020). Indeed, materials that have a low wetting ability or dry out easily are less susceptible to fungal degradation (Brischke and Alfredsen, 2020), as the Time of Wetness is short (Van Acker et al., 2014). The service life of wood-based building materials could consequently be increased by tailoring material moisture dynamics to the intended use or service class. From a sustainability perspective, such fit-for-purpose moisture dynamics are often easier achieved and preferred than wood protection by biocide preservative treatments. This approach is especially interesting with the advent of engineered wood products and wood fibre insulation materials, as there are many opportunities to alter material structure and moisture dynamics.

Understanding how material porosity and chemistry affect moisture dynamics is therefore essential for their efficient use, as well as for product optimisation. However, moisture characterisation is typically performed on solid wood and less is known about moisture behaviour of wood-based panels and wood fibre insulation materials. In this paper, we assess the moisture dynamics of three commonly used wood-based panels (radiata pine plywood, three-layer spruce panel and oriented strand board), thermally modified wood and six wood fibre insulation materials made using different manufacturing processes (wet and dry) and additives (bitumen, paraffin). Liquid water absorption and water vapour desorption is assessed with the floating test (CEN TS 16818 standard, 2018). While liquid water absorption is less relevant for insulation materials, which are usually applied in UC1, it was decided to include these materials to assess their suitability in applications with risk of condensation, leakage or rain water infiltration. Three complementary techniques are applied to elucidate the influence of material characteristics on the liquid water absorption and desorption behaviour of these materials: X-ray Computed Tomography (X-ray CT) to assess the porosity of the materials, Attenuated Total Reflectance Fourier Transform Infrared (ATR-FTIR) spectroscopy, to characterize the composition of the materials and Low-Field Nuclear Magnetic Resonance (LFNMR) spectroscopy, to determine the ‘water populations’ when the material is water saturated, which gives an indication of material porosity from macro voids to cell wall water. Based on these techniques, the impact of additives, manufacturing process and material structure on moisture dynamics and, consequently, service life is examined. More specifically, the following hypotheses are investigated: hydrophobic additives and thermal treatment reduce the amount and rate of water absorption, insulation materials with a higher porosity absorb more water and wood-based panels with macro pores absorb and retain more water. Additionally, the impact of the manufacturing process on the moisture dynamics of wood fibre insulation materials is assessed.

## Materials and Methods

### Wood-Based Materials

**Figure 1:**
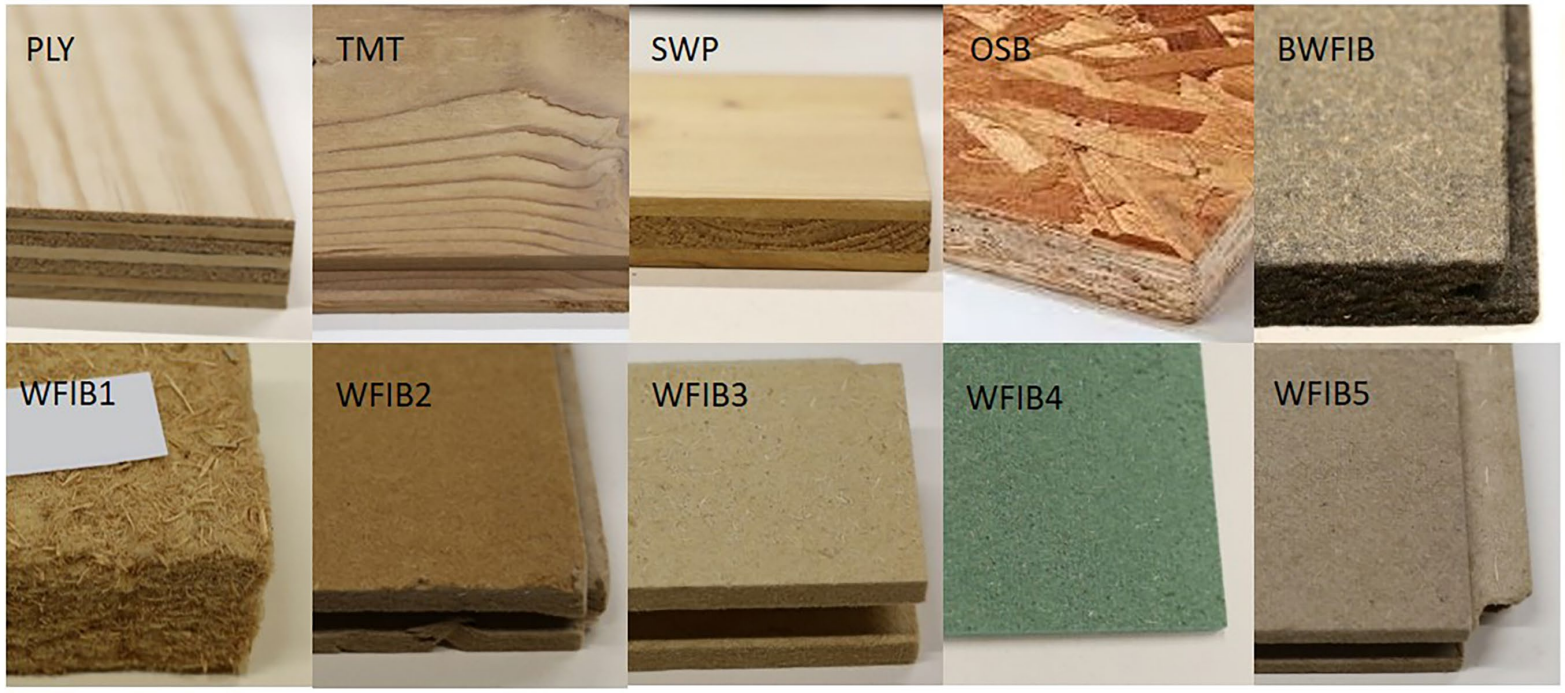
Overview of selected wood-based building materials. Radiata pine plywood (PLY), thermally modified spruce (TMT), three-layer spruce panel (SWP), oriented strand board (OSB), bituminised wood fibre board (BWFIB) and wood fibre insulation type 1–5 (WFIB1-5).

To have a broad view on the moisture dynamics of wood-based building materials in general, commercially available materials with different structures (solid wood, veneer-based, strand-based, and fibre-based), density, and additives (glue, paraffin, bitumen) were chosen (Table 1; Figure 1). Three commonly used wood-based panels [radiata pine plywood (PLY), three-layer spruce panel (SWP) and oriented strand board (OSB)], thermally modified spruce wood (TMT) and six wood fibre insulation materials (WFIBs) were used. The wood fibre insulation materials contain different additives, differ in density and were made using different manufacturing processes. The insulation materials purchased for this study were commercially produced with the following three manufacturing processes. In the wet manufacturing process for insulation boards, wood chips and shavings are broken down into fibers and mixed with water and additives, such as paraffin. This mix forms a continuous fibre cake, from which half of the water is removed with a mechanical press. The lignin in the wood fibres serves as a natural binding agent when heated with water (160–220°C), so no binding agent needs to be added. In the dry manufacturing process for insulation boards, the wood fibres are glued together with isocyanate adhesives. The adhesives are cured and hardened through exposure to a mixture of water vapour and air. The manufacturing process for flexible insulation mats is similar to the dry manufacturing process for insulation boards, though no water vapour is used, only hot air. Instead of isocyanates, polyolefine fibres are added, which partly melt due to the heat and make the wood fibres stick together to form a flexible wood fibre insulation mat.

**Table 1:** Overview of material components and/or treatment.

Label	Material	Components and/or treatment
PLY	Plywood radiata pine	Radiata pine veneers (2–3 mm thickness), glue (PF)
TMT	Thermally modified spruce	TMT process: 1) Hydrothermolysis up to 170°C 2) drying 3) heated again to up to 180°C in dry conditions without oxygen
SWP	Three-layer spruce panel	Spruce, isocyanate adhesive (bottom and top board 4.5 mm thick, middle board 9 mm thick)
OSB	Oriented strand board	Scots pine fibres, isocyanate adhesive
BWFIB	Wood fibre board with bitumen	Norway spruce/Scots pine fibres, bitumen emulsion
WFIB1	Wood fibre insulation 1 (mat)	Norway spruce/Scots pine fibres, ammonium phosphate, polyolefin fibres, dry production for flexible wood fibre insulation mats (hot air)
WFIB2	Wood fibre insulation 2 (board)	Norway spruce/Scots pine fibres, wet manufacturing process
WFIB3	Wood fibre insulation 3 (board)	Norway spruce/Scots pine fibres, isocyanate adhesive (4%), paraffin (4%), dry production for dimensionally stable and pressure-resistant boards (hot air and steam)
WFIB4	Wood fibre insulation 4 (board)	Norway spruce/Scots pine fibres, aluminium sulphate, paraffin (4%), dye, wet manufacturing process
WFIB5	Wood fibre insulation 5 (board)	Norway spruce/Scots pine fibres, paraffin (4%), wet manufacturing process

Table 2 provides the panel thickness (mm) and density (kg/m^3^) at 12% moisture content of the considered wood-based building materials, based on the technical data sheet(s). Additionally, pore volume estimations (%) based on the material density and based on the X-ray CT images are included, as well as the average moisture content after water saturation. The pore volume estimations were calculated as follows:


(1) $$V=1-\frac{D_{\mathrm{ma}t}}{D_{\mathrm{cell}}},$$

where *V* is the pore volume (%), *D*_mat_ the material density (kg/m^3^) and *D*_cell_ the density of cellulose (1,500 kg/m^3^). Since the wood species of the insulation materials was not specified, we opted to use cellulose density (Ioelovich and Laka, 2002).

**Table 2:** Overview of material properties: panel thickness (mm), density (kg/m^3^), pore volume (%) estimation based on the material density relative to a solid reference density of 1,500 kg/m^3^ (density of cellulose as reference value), pore volume (%) estimation of reference specimens (5 × 5 × 10 mm^3^) assessed with X-ray micro CT and the corresponding X-ray CT resolution (µm) and average moisture content (%) of LFNMR specimens (5 × 5 × 10 mm^3^) after water saturation under vacuum pressure.

Label	Panel thickness in dry state (mm)	Density (kg/m^3^)	Pore volume (%) estimation based on material density	Pore volume (%) estimation based on X-ray CT^*^	X-ray CT resolution (μm)	Average moisture content (%) LFNMR specimens after saturation
PLY	18	550	63	36	6.5	184 ± 9
TMT	18	380–450	70–75	29	6.5	247 ± 18
SWP	19	470	69	26	6.0	251 ± 93
OSB	18	600–680	55–60	45	6.0	136 ± 15
BWFIB	22	270	82	76	6.0	517 ± 38
WFIB1	25	50	97	92	6.0	762 ± 171
WFIB2	60	160	89	53	6.5	777 ± 57
WFIB3	40	140	91	80	6.0	579 ± 19
WFIB4	5	250	83	69	6.0	548 ± 81
WFIB5	40	160	89	x	x	x

* Latewood pores with a diameter smaller than the resolution were not visible with X-ray micro CT. x: not included in X-ray CT and LFNMR analysis.

### Floating Test

To assess the moisture behaviour of the selected wood-based materials, a floating test was performed according to CEN TS 16818 standard (2018). Specimens of 50 × 50 mm^2^ × panel thickness were prepared with a panel saw (Rema S.A.,Warsaw, Poland), with exception of the wood fibre insulation mat (WFIB1), which was prepared using scissors. The edges of the specimens (50 × 50 mm^2^ × panel thickness) were sealed with a solvent-borne polyurethane paint containing a polyisocyanate curing agent, mixed with a hardener, to prohibit water from entering through the sides (SigmaDur 520, PPG Industries). The specimens (five replicates per material) were conditioned (65% RH, 20°C) for 2 weeks and weighed (*m*_i_) with an accuracy of 10^−4^ g (Sartorius A200S, Sartorius Lab Instruments GmbH & Co. KG, Goettingen, Germany). Then, inside the conditioning room (65% RH, 20°C), specimens were put on the water surface of containers filled with deionized water. The specimens were blotted on a wet cloth and weighed (*m*_t_) at several time intervals (5, 30 s, 1, 10, 5, 10, and 30 min, 1, 4, 8, 24, 48, 72, and 144 h). Some of the selected materials were expected to absorb water very fast, so a high temporal resolution was chosen at the beginning of the experiment. After 144 h of absorption, the materials were put on drying racks in a conditioning room and were weighed again after 1, 4, 8, 24, 48, 96, and 168 h to assess the desorption rate. After 168 h of desorption, the specimens were oven dried at (103 ± 2) °C and weighed again (*m*_0_).

The water uptake *W* (g/cm^2^) was calculated over time, using Eq. (2), with *A* (cm^2^) being the test surface area of the specimen, *m*_t_ (g) the mass of the specimen and *m*_i_ (g) the initial mass of the specimen:


(2) $$W=\frac{m_{t}-m_{i}}{A}.$$

The materials were classified in absorption classes, based on the water uptake (Table 3).

**Table 3:** Classification of absorption based on water uptake and release (g/cm^2^) after 144 h, adapted from Van Acker et al. (2014).

Class	Maximal water uptake (g/cm^2^) after 144 h of absorption
1	0.75
2	0.95
3	1.15
4	1.35
5	1.75
6	2.75
7	5.00
8	>5.00

Absorption and desorption curves were fitted (non-linear least-squares fit, lmfit package, Python) to the water uptake (g/cm^2^) datapoints based on Eqs. (3) and (4), respectively, similar to Van Acker et al. (2014):


(3) $$f\left(t\right)=at^{b},$$

(4) $$f\left(t\right)=a+be^{-\frac{t}{c}},$$

where *a*, *b*, and *c* are curve-fitting parameters. The residual moisture (*ω*_r,t_) after *t* hours of desorption was calculated as follows:


(5) $$\omega_{r,t}=\omega_{i}-\omega_{t},$$

with *ω*_i_ (%) the initial moisture content and *ω*_t_ (%) the moisture content after *t* hours of desorption.

The bottom layer of the wood fibre boards manufactured with the wet manufacturing process, is denser and harder than the rest of the board. In an additional floating experiment, five specimens of WFIB2 and WFIBF5 were therefore put afloat with the hard bottom side and five with the soft top side in water contact. Additionally, the influence of potential defects in the hard bottom side was assessed by drilling a bore hole of 5 mm diameter and 5 mm deep for five additional specimens of WFIB2 and WFIBF5. Although WFIB4 was also manufactured with the wet manufacturing process, it was excluded from these experiments because of its limited panel thickness of 5 mm.

### Low-Field Nuclear Magnetic Resonance Measurements

Low-Field Nuclear Magnetic Resonance (LFNMR) spectroscopy is a technique used to assess the realignment of hydrogen nuclei spins in a permanent magnetic field after spin orientation has been changed by a radio frequency pulse. The realignment is described by the relaxation time, and the technique is particular well-suited for fluids confined in solid materials, e.g., water molecules in wood (Fredriksson and Thygesen, 2017). The relaxation time depends on how strongly the water interacts with the solid material, which depends on the chemical and physical environment in which the water molecule is situated. The spin–spin relaxation time or *T*_2_ value of water molecules in smaller pores is shorter than in larger pores, when the pore wall surfaces are similar (Fredriksson and Thygesen, 2017). Consequently, LFNMR can be applied to gain knowledge on water populations in different environments within a material.

Specimens of 5 × 5 × 10 mm^3^ were prepared with a razor blade. Five replicates for each material were water saturated under vacuum and stored in water-filled sample tubes. The specimens were put in a pre-weighed glass LFNMR tube, after the excess surface water was removed by applying a wet cloth without drying the specimens. The wet weight of the specimen was measured with an accuracy of 10^−4^ g (QUINTIX224-1S, Sartorius Lab Instruments GmbH & Co. KG, Goettingen, Germany). The average moisture content of the LFNMR specimens after water saturation is listed in Table 2. A solid Teflon rod was inserted into the glass LFNMR tube to fill the remaining space, thereby limiting water evaporation from the specimen during the experiment. The glass LFNMR tube containing the specimen was placed in a Bruker mq20 minispec with a 0.47 T permanent magnet (Bruker, Billerica, MA, United States) to perform LFNMR measurements. The temperature in the LFNMR around the specimen was kept constant at 22°C by circulation of water in the probe using a Julabo Refrigerated and Heating Circulator (Julabo GmbH, Seelbach, Germany). The specimen was allowed to thermally equilibrate in the instrument for 2 min before starting each measurement.

The Carr-Purcell-Meiboom-Gill (CPMG) pulse sequence was used to measure the spin–spin relaxation time (*T*_2_) of the specimens with a pulse separation (*τ*) of 0.1 ms, 8,000 echoes, 32 scans and a recycle delay of 30 s. Exponential decay analysis (Istratov and Vyvenko, 1999) was applied to the recorded LFNMR decay curves to give smooth, continuous distributions of *T*_2_ relaxation times. The range for the distribution was set to 0.2–2,500 ms for wood-based panels and 0.2–4,200 ms for wood fibre insulation materials. The latter range was necessary for fibre-based insulation materials, since the water-saturated specimens contained much more water and exhibited slower relaxation of the LFNMR signal. For each peak, the relative sum of amplitudes of the exponential components as well as their *T*_2_ values corresponding to maximum peak intensity were determined. The Kruskal–Wallis H-test, a nonparametric statistical test, was applied for testing whether the median *T*_2_ values and median relative peak sum of amplitudes of the assessed wood-based materials were different. When the difference between medians was significant, Dunn’s multiple comparison test (Dunn, 1961) was applied to pinpoint for which materials the medians were different. Benjamini-Hochberg correction (Benjamini and Hochberg, 1995) was performed to control the false discovery rate (Type I error).

### Attenuated Total Reflectance Fourier Transform Infrared Spectroscopy

To assess the effect of the manufacturing process on the composition of the wood fibre insulation materials, Attenuated Total Reflectance Fourier Transform Infrared (ATR-FTIR) spectroscopy was applied. In ATR-FTIR, a beam of infrared light passes through a crystal on which a sample is positioned (Djajadi et al., 2017). Depending on the interaction with the sample and the wavenumber, an FTIR absorbance spectrum is obtained, providing information on the presence of certain functional groups, such as CH₂, OH, and C=O. Each material was ground to a powder with a particle size smaller than 0.1 mm using a centrifugal mill (ZM 200, Retsch GmbH, Haan, Germany). The powder was dried in a BINDER VD23 vacuum oven (BINDER GmbH, Tuttlingen, Germany) for 24 h at 60°C. For each material, three ATR-FTIR measurements were performed on the oven-dried powders using a Nicolet 6,700 FT-IR, Pike Technologies GladiATR diamond spectrometer (Thermo Scientific, Waltham, MA, United States), with a working temperature of 25°C. The spectral range included was 3,700–500 cm^−1^ and spectra were obtained using 64 scans (128 for the background) and a resolution of 2.0 cm^−1^. Atmospheric correction was applied in OMNIC (version 9.8.372, Thermo Fisher Scientific Inc.). For each of the three replicates, linear baseline correction was applied on 13 individual peak regions. A baseline-corrected spectrum was obtained for each replicate and normalized by dividing all absorbance values by the absorbance value of the highest peak (ca. 1,026 cm^−1^). After smoothing with a Savitzky–Golay filter (polynomial = 3, window = 9), a mean spectrum was obtained for each material (Supplementary Figure 1).

Principal component analysis was applied to determine whether the manufacturing process (wet or dry, using heat or steam) and/or the additives (paraffin, bitumen emulsion and polyolefins) had a significant effect on the FTIR spectra of wood fibre insulation materials. Principal component analysis was performed on the smoothed FTIR spectra of the replicates in MATLAB version R2021a (Mathworks Inc., Natick, MA, United States) to determine which IR absorbance bands differed between the materials.

### X-Ray Computed Tomography

X-ray Computed Tomography (X-ray CT) was used to obtain a 3D visualisation of the internal material structure and to determine the pseudo-pore distributions of the wood-based materials. For PLY, TMT, SWP and OSB, one of the specimens (5 × 5 × 10 mm^3^) previously exposed to LFNMR was selected. To prevent cracks, the water-saturated specimens were allowed to partially dry in laboratory climate before oven drying, which was necessary to determine the moisture content at water saturation. For BWFIB, WFIB1, WFIB2, WFIB3, and WFIB4, water saturation under vacuum pressure and subsequent drying after the LFNMR measurement had altered the internal structure of the specimens considerably. Therefore, new specimens were selected for X-ray CT scanning of these materials. One specimen per material (5 × 5 × 10 mm^3^) was put on a sample holder and scanned with the Nanowood scanner (Dierick et al., 2014) at the Centre for X-ray Tomography at Ghent University (UGCT).^1^ All specimens were scanned at an average voltage of 50 kV, a target current of 100 μA, and an exposure time of 1,000 ms per image, resulting in an approximate scan time of 38 min per specimen obtaining 2001 projections. Reconstruction was performed using Octopus (Vlassenbroeck et al., 2007), a tomography reconstruction package for parallel and cone-beam geometry. The resulting high-resolution scans had an approximate voxel pitch of 6–6.5 μm (Table 2).

Reconstructed images of the specimens were visualized and analyzed using Octopus Analysis (Brabant et al., 2011). Due to the scan resolution and the specimen size, this set-up allowed for assessment of pores with a minimum radius of approximately two times the resolution (due to partial volume effects) and a maximum radius of 11.2 mm. A bilateral filter was applied on the reconstructed images, followed by thresholding, resulting in a binary image volume, with white voxels representing the wood mass and black voxels representing the pores. The minimum pore size analysed was 1 voxel. From this binary image volume, the pore volume (%) was inferred (Table 2). Due to the scan resolution, part of the latewood pores was obscured after thresholding, resulting in significant differences with the pore volume estimation based on density. This discrepancy was the highest for materials that did not have macro voids, such as plywood, modified spruce and three-layer spruce panel, as a large part of their pore volume is comprised of small latewood pores. The total pore volume based on CT is thus an approximation of all pores that are at least larger than 6.5 μm.

By consecutive expanding and shrinking of the thresholded volume in Octopus Analysis, a pseudo-pore size distribution was acquired (Supplementary Figure 2). The relative pore volume was calculated as follows:


(6) $$V_{r,n}=\frac{V_{n}\;}{V_{\mathrm{tot}}\;},$$

with *V_r,n_* (%) the relative pore volume of the pores that were filled by *n* consecutive expanding and shrinking steps, *V_n_* (voxels) the pore volume after *n* expanding steps followed by *n* shrinking steps and *V*_tot_ the total pore volume (voxels) when no expansion or shrinking had been applied.

**Figure 2:**
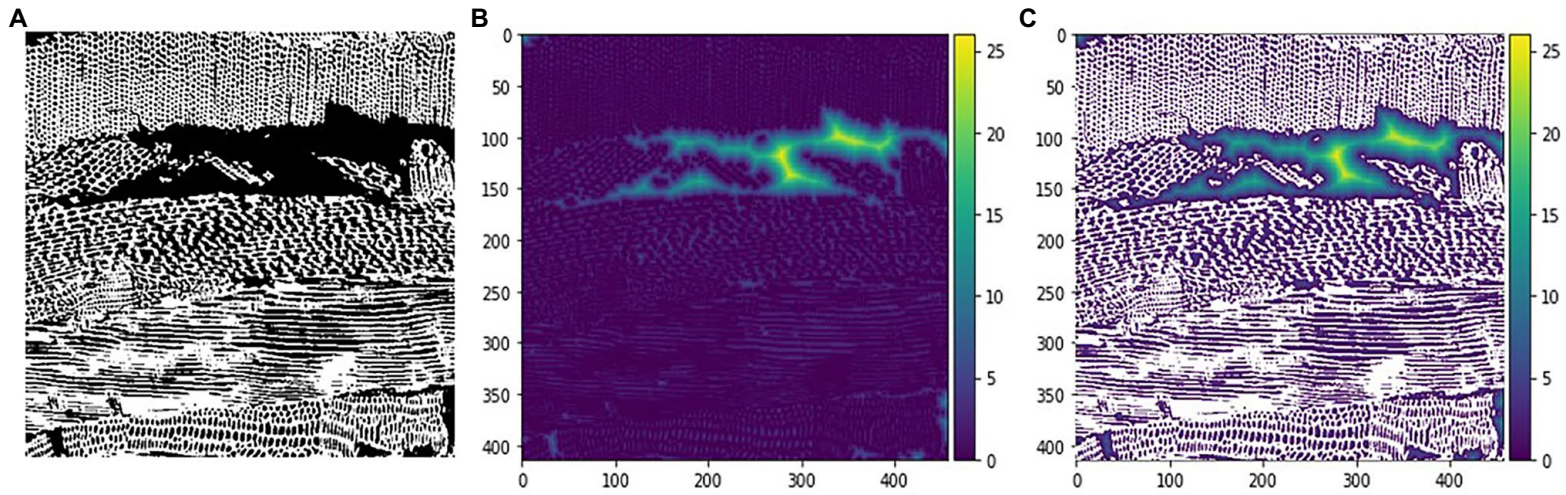
Example of Euclidean distance transform on an X-ray CT image of OSB. **(A)** Binary image **(B)** result Euclidean distance transform **(C)** result Euclidean distance transform visualised on top of the binary image. The colour bar on the right-hand side indicates the Euclidean distance in voxels.

The Euclidean distance from pore centre to pore wall was computed in Python (Python Software Foundation)^2^ based on the thresholded binary image volume. The Euclidean distance from every pore voxel to the nearest pore wall was calculated in 3D with the Euclidean distance transform function in the multidimensional image transform package (package: scipy.ndimage, function: distance_transform_edt; Figure 2). Additionally, skeletonization was applied on the binary image volume in 3D, to assess the location of the centre voxel(s) of each pore (package: skimage.morphology, function: skeletonize_3d). Note that for a perfect sphere, the centre of the pore is represented by one voxel, while for a cylindrical pore the centre is represented by a line. To determine the Euclidean distance from pore centre to pore wall, the Euclidean distance transform matrix was multiplied with the skeletonization matrix, so that only the Euclidean distance from the pore centres to the pore walls was listed. A pseudo-pore distribution based on the Euclidean distance from pore centre to pore wall was determined by dividing the occurrence of each Euclidean distance by the total amount of assessed Euclidean distances from pore centre to pore wall.

## Results and Discussion

### Characterization of Water Populations and Pore Distribution With LFNMR and X-Ray CT

#### Wood-Based Panels

**Figure 3:**
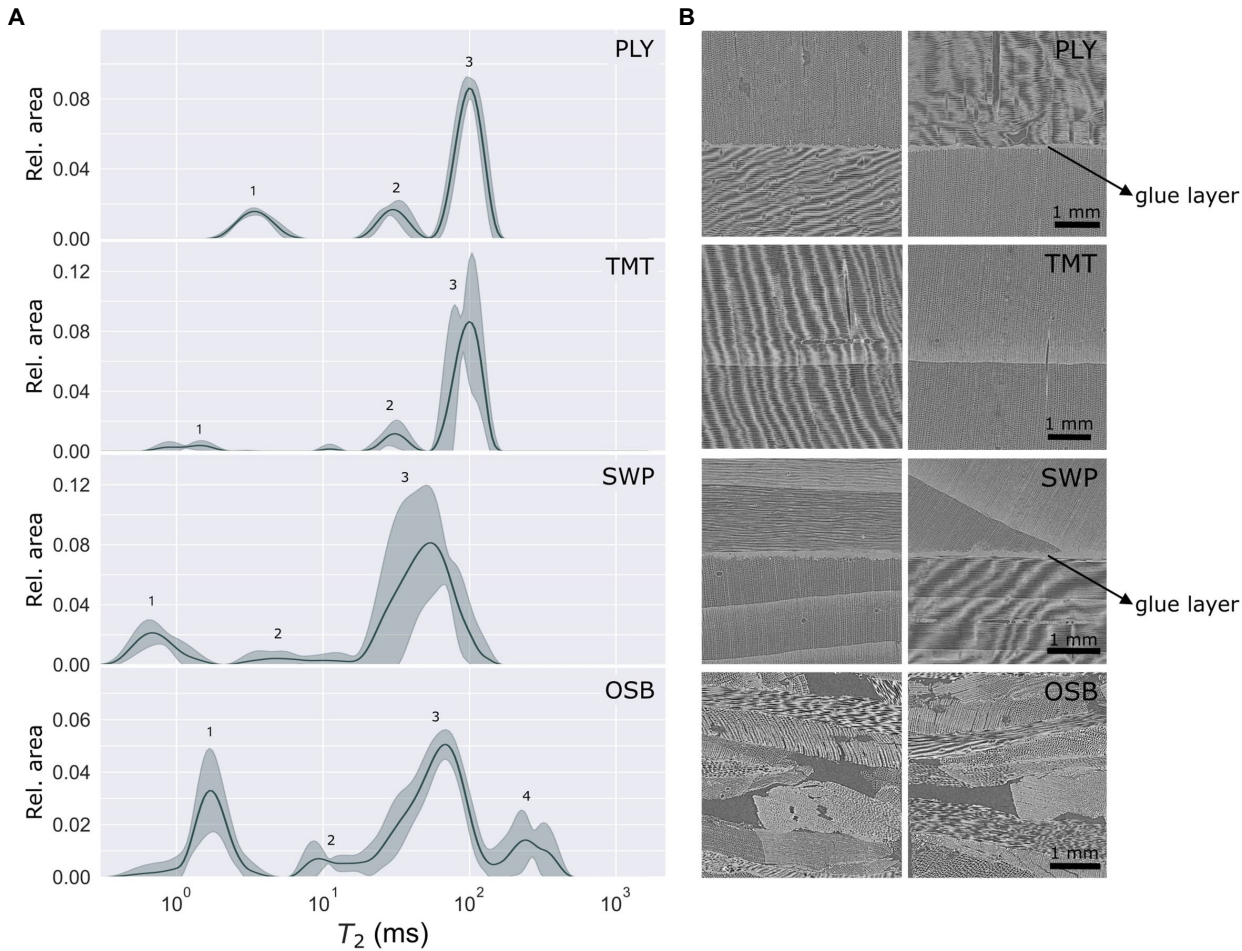
**(A)** Continuous *T*_2_ distributions (with peak numbers 1, 2, 3 and 4) of four wood-based panels, showing the mean value (line) and standard deviation (filled zone); **(B)** X-ray CT section of the wood-based panels, appr. 4 × 4 mm^2^, showing the center of the specimen seen from the front (left) and the side (right) for each specimen (5 × 5 × 10 mm^3^). Radiata pine plywood (PLY), thermally modified spruce (TMT), three-layer spruce panel (SWP) and oriented strand board (OSB).

The water populations of each material were assessed with LFNMR in water-saturated state. The spin–spin relaxation time (*T*_2_) indicates how closely the water is interacting with the solid material. For untreated softwood, usually three peaks are observed: the 1st peak related to cell wall water at *T*_2_ < 4 ms (Fredriksson and Thygesen, 2017; Beck et al., 2018), the 2nd peak related to free water located in pits and the 3rd peak related to water in tracheid lumina (*T*_2_ > 40 ms). The thermally modified wood and wood-based panels (Figure 3A) showed this typical distribution pattern, apart from OSB, which had an additional peak at 262.8 ms. Peak 1, peak 2 and peak 3 *T*_2_ values of radiata pine plywood agreed to those found for untreated radiata pine in a previous study by Beck et al. (2018) (Supplementary Table 1). The relative sum of amplitudes for each peak (i.e., peak areas) was of the same order of magnitude as well (Supplementary Table 2). The plywood manufacturing process did not seem to have any substantial influence on pore distribution and pore surface hydrophobicity. Indeed, in the X-ray CT images (Figure 3B), only a very small glue layer could be observed in-between intact radiata pine layers, with no excess of glue filling up pores in the proximity of the glue layer. Similarly, the *T*_2_ times of peak 2 and 3 of the three-layer spruce panel specimens are of the same order of magnitude as those found by Fredriksson and Thygesen (2017) for solid Norway spruce, as well as the relative sum of amplitudes. Thus, when the wood anatomy of a species remains intact, the water populations of the wood-based panel were not affected in the water-saturated state.

The LFNMR relaxation time distributions of oriented strand board (OSB) specimens, however, contained a fourth peak. In previous LFNMR studies, peaks in this region were attributed to surface water on the wood specimens (Fredriksson and Thygesen, 2017; Beck et al., 2018). However, properly removing excess water from all sides of the specimen with a wet cloth should avoid peaks related to surface water. As the fourth peak was absent in the other wood-based panel specimens, its consistent presence in the OSB specimens was therefore a clear indication of water in pores larger than tracheid lumina. With X-ray CT, pores with Euclidean distances from centre to pore wall of 12–150 μm could indeed be observed between the wood strands. From the X-ray CT images, it could be observed as well that the wood anatomy of the separate strands had remained relatively intact, with the tracheids being clearly discernible (Figure 3B).

**Figure 4:**
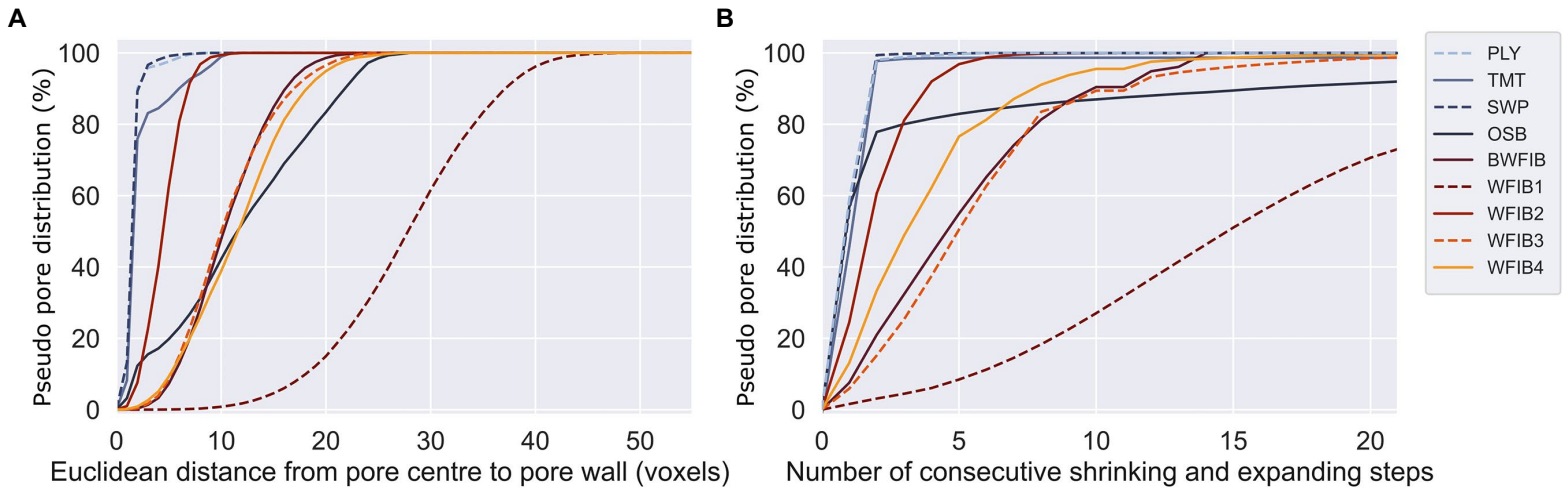
Cumulative representation of pseudo-pore distributions (X-ray micro CT at 6–6.5 μm resolution) of one reference specimen per material based on **(A)** Euclidean distance – skeletonization approach and **(B)** on shrink and expand approach. PLY, Plywood radiata pine; TMT, Thermally modified spruce; SWP, Three-layer spruce panel; OSB, Oriented strand board; BWFIB, Bituminised wood fibre board; WFIB1-4, Wood fibre insulation board type 1–4.

For wood-based panels, there was a strong correlation between the LFNMR data and the pseudo-pore distributions assessed with X-ray CT (Figure 4). OSB clearly differed from plywood (PLY), three-layer spruce panel (SWP) and thermally modified spruce (TMT). For the latter three wood products, 99% of the pores were filled after 2 consecutive shrinking and expanding steps (Figure 4B). Since pits (peak 2) cannot be observed at a scan resolution of 6–6.5 μm, the pore distributions of PLY, SWP and TMT corresponded to the LFNMR tracheid peak (peak 3). Consequently, it can be assumed that the pores filled after 2 consecutive shrinking and expanding steps in OSB likewise corresponded to peak 3. Indeed, 82% of the combined relative area of peaks 3 and 4 was represented by peak 3, corresponding well to the amount of pore volume (78%) covered by pores that were filled after 2 consecutive shrinking and expanding steps (Figure 4).

#### Thermally Modified Wood

Wood modification changes the hydrophobicity of the pore surface and has been shown to significantly affect LFNMR relaxation time distributions by causing shifts in *T*_2_ times (Digaitis et al., 2021). Acetylation has been shown to cause an increase in *T*_2_ up to about 25 ms for peak 2 and up to about 100 ms for peak 3 for radiata pine wood, depending on the degree of acetylation (Beck et al., 2018). Here, thermally modified Norway spruce showed three peaks, with an increased *T*_2_ time for peaks 2 and 3 as compared to the LFNMR relaxation time distributions assessed by Fredriksson and Thygesen (2017). The mean *T*_2_ value of peak 2 was significantly higher than that of untreated Norway spruce. Peak 3 had a mean *T*_2_ value of 97.5 ms, which, although high, remained in the range of *T*_2_ values previously reported for earlywood of Norway spruce (57.6–103.8 ms). However, since the X-ray CT-images showed a clear presence of both earlywood and latewood (45.4–77.4 ms), the increased *T*_2_ value of peak 3 was most likely due to thermal modification. These results are in accordance with the study of Cai et al. (2020), in which the *T*_2_ values of peaks 2 and 3 had increased for thermally modified spruce. Likewise, thermal modification has been shown to increase the *T*_2_ values of peaks 2 and 3 in radiata pine, Chinese fir, loblolly pine, Scots pine and European ash (Guo et al., 2018; Gao et al., 2019; Wang et al., 2019; Cai et al., 2020). The following hypotheses have been put forward to explain the increase in *T*_2_ values: increase in lumen diameter due to removal of extractives and resin and deformation and merging of lumen tracheids, a rougher lumen surface due to microcracking and partial elimination of hydroxyl groups due to degradation of hemicelluloses (Guo et al., 2018; Cai et al., 2020).

#### Wood Fibre Insulation Materials

**Figure 5:**
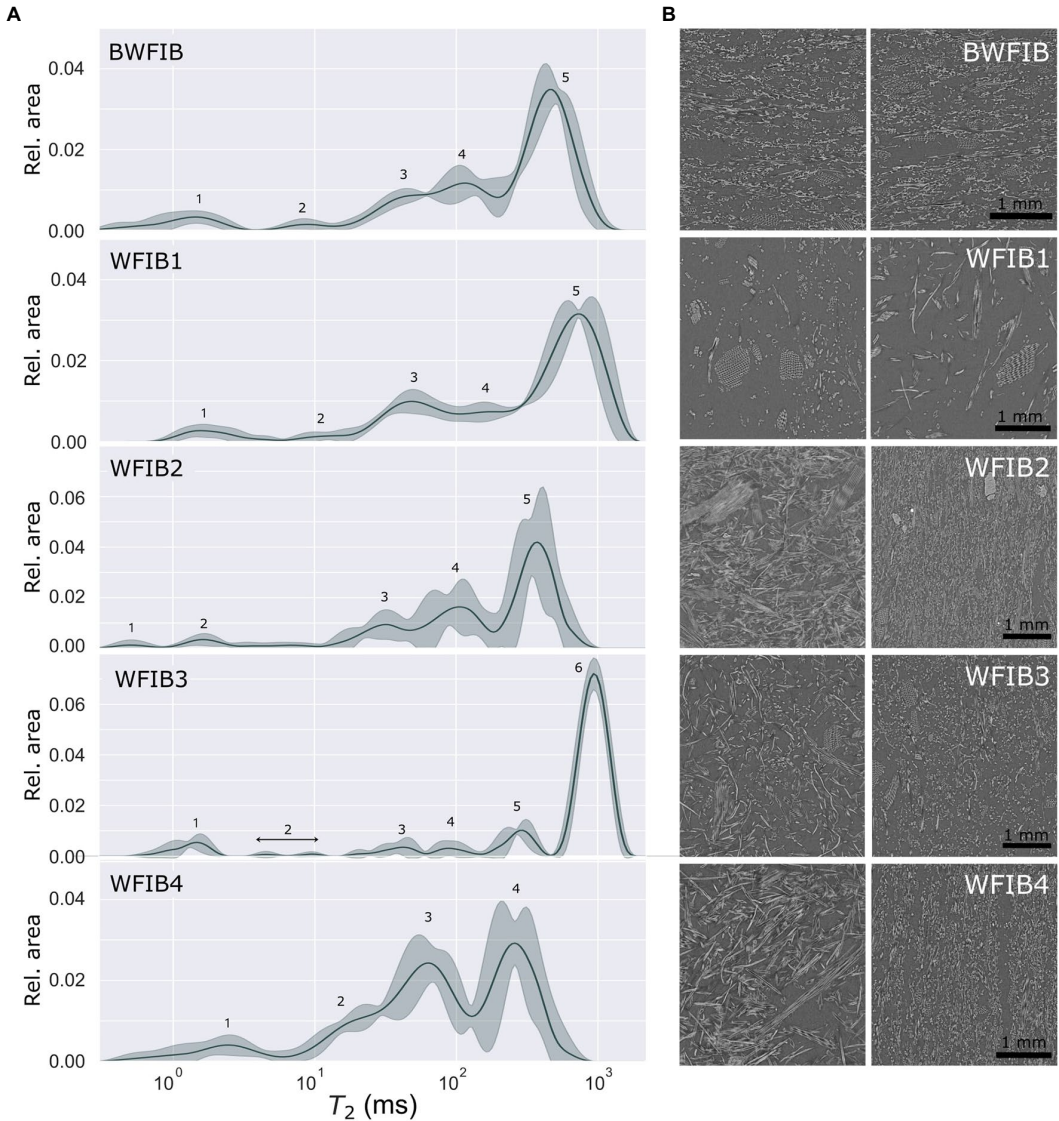
**(A)** Continuous *T*_2_ distributions (with peak numbers 1–6) of five wood fibre insulation materials, showing the mean value (line) and standard deviation (filled zone); **(B)** X-ray CT section of the wood fibre insulation boards, appr. 4 × 4 mm^2^, showing the center of the specimen seen from the front (left) and the side (right) for each specimen (5 × 5 × 10 mm^3^). BWFIB = Bituminised wood fibre board and WFIB1-4 = Wood fibre insulation board type 1–4.

The LFNMR relaxation time distributions of the wood fibre insulation materials generally contained five peaks, with the exception of WFIB4, which had four and WFIB3, which had six (Figure 5A). The peaks were less distinctive than those of the wood-based panels, with overlapping peaks in the region of 10 ms and higher. Since these materials consist of wood fibres, the first two peaks were expected to correspond to peak 1 at a *T*_2_ < 4 ms (cell wall water) and peak 2 (water in pits). The mean *T*_2_ values for peaks 1 and 2 were indeed in the range expected for Norway spruce. On the X-ray CT-images, loosely connected wood fibres were clearly discernable in the porous wood fibre insulation materials (Figure 5B), especially when scrolling through the virtual 3D volume. Since intact tracheid structures occurred across the volume, we would expect a peak in the range of the *T*_2_ values occurring for tracheid lumen in Norway spruce (45–104 ms) as well. For WFIB1 and WFIB4, this appeared to be the case. For WFIB2, WFIB3 and BWFIB, peak 3 was located at a *T*_2_ value lower than the range for solid Norway spruce wood (Supplementary Table 1; Fredriksson and Thygesen, 2017). Either the tracheid size of the wood fibres had decreased, for instance due to compression during the manufacturing process, or the shifts in *T*_2_ values were caused by the presence of additives. Although WFIB3 and BWFIB contain hydrophobic additives in the form of paraffin and bitumen, respectively, it is unlikely that the tracheid lumen size is decreased by the tracheid lumens being filled with aforementioned additives, as the peak 3 location for WFIB3 and BWFIB was similar to WFIB2. In a study on solid pine wood, *T*_2_ values were little affected by wax treatment (Wang et al., 2019). Consequently, tracheid deformation due to compression is more likely.

Peaks 4, 5 and 6 do not occur in LFNMR relaxation time distributions of solid Norway spruce and Scots pine (Fredriksson and Thygesen, 2017; Beck et al., 2018). Since they were located on the right-hand side of the tracheid peaks, their clear and consistent presence in the LFNMR relaxation time distributions of wood fibre insulation materials was related to water located between wood fibres (tracheid bundles). The wood fibre insulation materials were indeed highly porous, with porosities ranging from 82 to 97% (based on density) and 53–92% (based on X-ray CT-images), see Table 2. Pseudo-pore distributions were assessed based on X-ray CT images of each material (Figure 4), of which cross sections are shown in Figure 5B. Unlike OSB, the relative pore volumes of pores of similar size as tracheid lumens as assessed by X-ray CT, did not correspond well with the ones estimated with LFNMR. In contrast to wood-based panels, which have a more rigid matrix and lower macro porosity, the pore structure of wood fibre insulation materials changed significantly during water absorption. Consequently, the pore size distributions derived from X-ray CT images of dry wood fibre insulation specimens were unfit for comparison with the LFNMR spectra of water-saturated specimens. A distinct example is WFIB1, which is composed of loosely connected wood fibres with an overall porosity of 97% and a density of 50 kg/m^3^. During water absorption, the fluffy nature of the material is lost and the wood fibres were in closer contact, eliminating part of the pores that were present in dry state. Instead of WFIB1, which had the largest pores in dry state (Figure 4), WFIB3 had a peak with the largest *T*_2_ value (Figure 5B). It can also be noted that, although the pseudo-pore distributions of BWFIB, WFIB3 and WFIB4 were very similar in the dry state, their LFNMR relaxation time distributions differed significantly (Figure 5A; Supplementary Table 1). Although the X-ray CT images could not be used to provide reliable estimates of the pseudo-pore volume in water-saturated state, the images were useful for detecting intactness of the original wood fibres.

The LFNMR relaxation time distributions of WFIB1, WFIB2 and BWFIB were similar. They all contained five peaks and the *T*_2_ values and relative areas under each peak did not differ significantly based on the Kruskal–Wallis H-test with Dunn’s multiple comparison test as post-hoc test. Even though the water-saturated BWFIB specimens had an average moisture content of 517%, being more than 200% lower than that of WFIB1 and WFIB2 (Table 2), the water populations experienced similar constraints in all three wood fibre materials. This difference in moisture content at water saturation can be attributed to the bitumen fraction in BWFIB, clearly inducing hydrophobicity, thereby reducing the water-holding capacity. The additional peak (peak 6) for WFIB3 at the highest *T*_2_ value could be an indication of a more rigid matrix, as the pore structure of WFIB3 remained more intact during water saturation than the other wood fibre insulation materials, which had similar pore distributions in the dry state (Figure 4). Presumably, this rigidity was caused by the dry manufacturing process with isocyanate glue. Although WFIB3 contained the largest pores, its moisture content at water saturation was, similar to BWFIB, about 200% lower than that of WFIB1 and WFIB2 due to the hydrophobic components (Table 2). Note that the moisture content is not the mass fraction of water, but the ratio of water mass (total mass of water in the wood) to wood mass (the dry mass of the wood alone). The moisture contents of the water saturated WFIB specimens were all more than 100%, as the water mass was greater than the wood fibre mass.

### Composition of the Wood-Based Materials Assessed With ATR-FTIR

**Figure 6:**
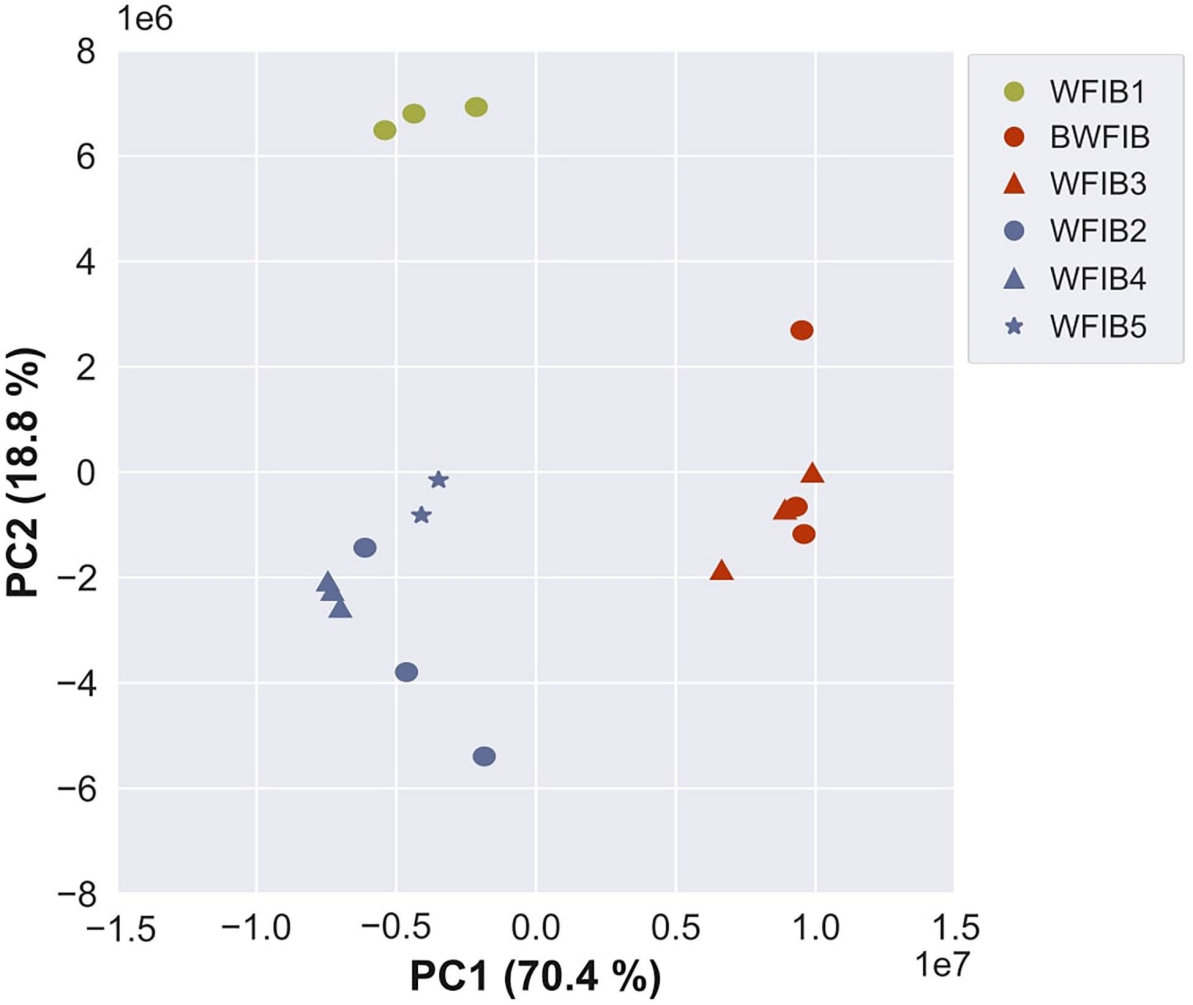
PCA of the FTIR spectra (3,500–500 cm^−1^ region) of 6 wood fibre insulation boards: score plot of PC1 (70.4%) vs. PC2 (18.8%). BWFIB = Bituminised wood fibre board and WFIB1-5 = Wood fibre insulation board type 1–5.

**Figure 7:**
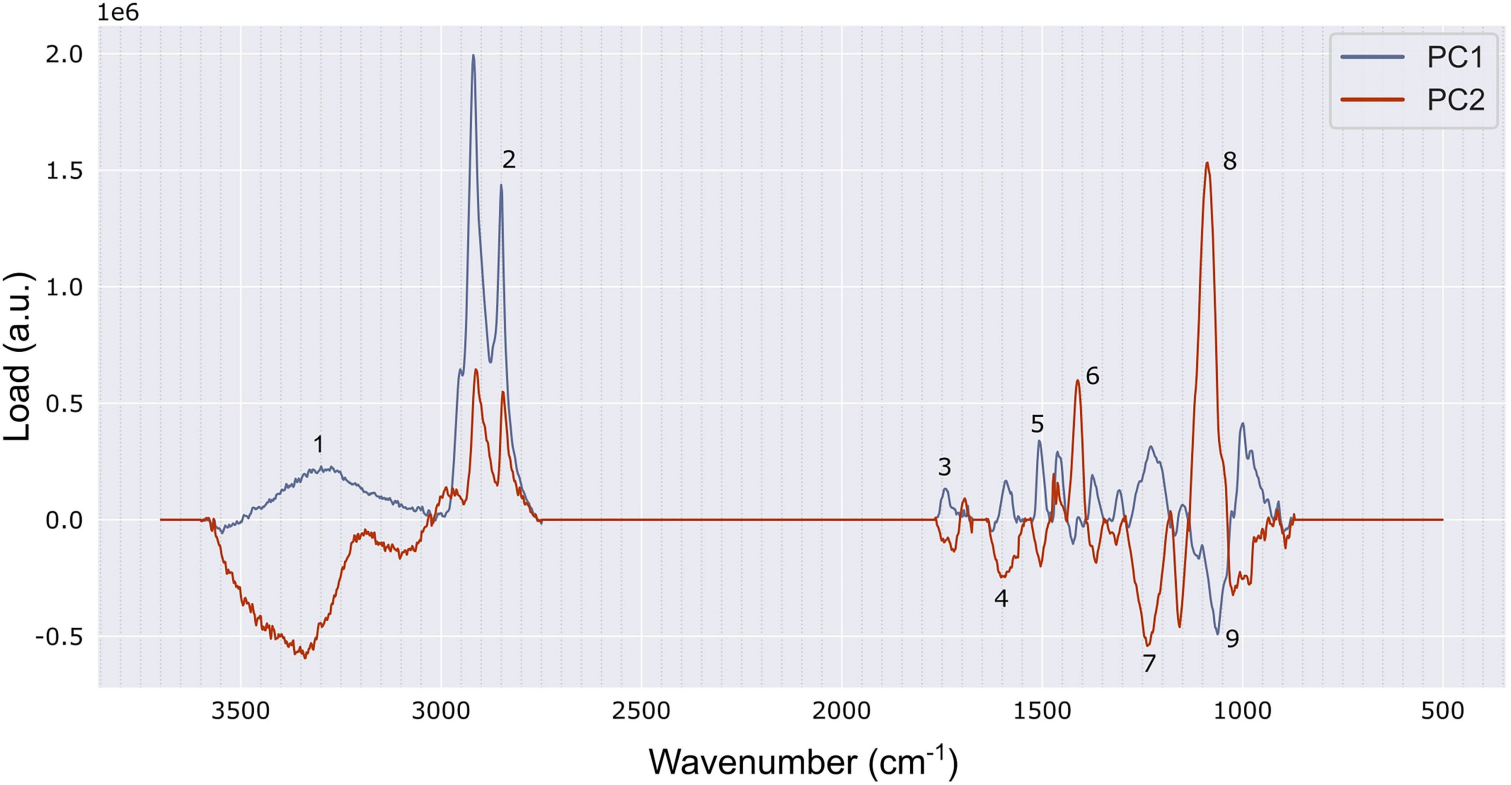
Loading profiles of PC1 and PC2, with the bands that mostly contributed to the separation highlighted. (1) 3,300 cm^−1^ assignable to OH stretching, (2) 2,848 and 2,918 cm^−1^ associated with CH_2_ stretching, (3) 1739 cm^−1^ related to stretching vibrations in the O=C–OH group, for example of the glucuronic acid unit in the xylan (Guo et al., 2018), (4) 1,603 cm^−1^ related to aromatic skeletal vibrations in lignin, (5) 1,507 cm^−1^ related to asymmetric aryl stretching in lignin, (6) 1,412 cm^−1^ related to HCC, HCO and HOC bending in cellulose, (7) 1,256 cm^−1^ (observed at 1,240 cm^−1^) tentatively assigned to CO stretching in lignin, (8) 1,088 cm^−1^ related to COC, C–C and ring vibration in non-cellulosic polysaccharides (NCPs) and (9) 1,063 cm^−1^ related to CC and CO stretching in cellulose (assignments based on Lupoi et al., 2015).

The wood fibre insulation materials were classified in 3 groups based on principal component analysis of the FTIR spectra: (1) BWFIB and WFIB3, (2) WFIB1 and (3) WFIB2, 4 and 5 (Figure 6). PC1 (70.4% of variance) and PC2 (18.8% of variance) indicated for which wavenumber regions the spectra of BWFIB & WFIB3, and WFIB1, respectively, were found to differ from the other spectra (Figure 7). Both WFIB1 as well as BWFIB & WFB3 showed distinctly higher peaks at wavenumbers 2,918 cm^−1^ and 2,848 cm^−1^, indicating an increased presence of alkanes (Kristensen et al., 2008; Khanifah et al., 2018) as compared to the other wood fibre insulation materials [Figure 7 (2)]. Since the wood fibres used in the wood fibre insulation materials originated from the same source, the absorbance increase is most probably the result of the hydrophobing agents (Table 1), being bitumen for BWFIB, polyolefin fibres for WFIB1 and paraffin for WFIB3. Indeed, paraffin consists of alkanes and FTIR spectra of pure paraffin have their main peak at wavenumbers 2,917–2,918 cm^−1^ and 2,848 cm^−1^ (Khanifah et al., 2018). Polyolefin fibres are made of at least 85% ethene, propene or other olefin units, which consist of CH_2_ and CH bonds (Mather, 2009). Similarly, bitumen contains a considerable number of alkanes. Since alkanes are hydrophobic compounds, a high abundance of these compounds is expected to increase the overall hydrophobicity of these materials. A higher absorbance for CH and CH_2_ stretching indeed corresponded to a lower moisture content in saturated state for BWFIB and WFIB3 (Table 2). However, this was not the case for WFIB1. Clearly, the nature and distribution of the hydrophobic components are important. Bitumen and paraffin cover part of the wood fibres, reducing the total area of available cell wall polymer surface and thereby also part of the water-binding sites (Lozhechnikova et al., 2015). It is unlikely that polyolefin fibres, being loose hydrophobic fibres, cover the wood fibres in such a way that water-binding sites cannot be reached. Interestingly, the technical data sheets of WFIB4 and 5 also indicated the addition of 4% paraffin, but this did not cause such an increased peak as for WFIB3. Likely, the wet manufacturing process of the latter materials hindered a homogenous distribution and binding of paraffin with the wood fibres over the entire wood fibre board, so that these were not present in the wood powder that was analysed. Indeed, the PCA analysis grouped WFIB2 (without additives) together with WFIB4 and WFIB5.

WFIB1 showed increased absorbance at 1,412 cm^−1^ and 1,088 cm^−1^, related to cellulose and hemicellulose bonds [Figure 7 (6,8)] and decreased absorbance at 1,603 cm^−1^, 1,507 cm^−1^ and 1,240 cm^−1^, related to lignin [Figure 7 (4,5,7)]. WFIB1 is the only insulation material manufactured with hot air, in contrast to the dry manufacturing process of WFIB3, in which a mixture of hot air and steam is used, and the wet manufacturing process of WFIB2, 4 and 5, in which wood fibres have been immersed in water before heating. In hydrothermal treatments, hemicelluloses have been shown to be strongly degraded from the beginning, in contrast to thermal treatments for which the main degradation acts on lignin in the initial stage of thermal degradation (Marcon et al., 2021). Consequently, this higher abundance of cellulose and hemicellulose bonds in WFIB1 as compared to the other wood fibre insulation materials, is likely related to the absence of water in its manufacturing process. The spectra were normalized by dividing by the absorbance of the highest peak, being 1,026 cm^−1^ related to CC and COH in non-cellulosic polysaccharides (NCPs), including hemicelluloses (Lupoi et al., 2015). We conjecture that the lower absorbance for OH binding in the region of 3,600–3,200 cm^−1^ in WFIB1 was caused by this reference NCPs peak being higher.

### Relating Water Absorption and Desorption Properties to Material Characteristics

#### Impact of Overall Porosity on Water Absorption and Desorption

**Figure 8:**
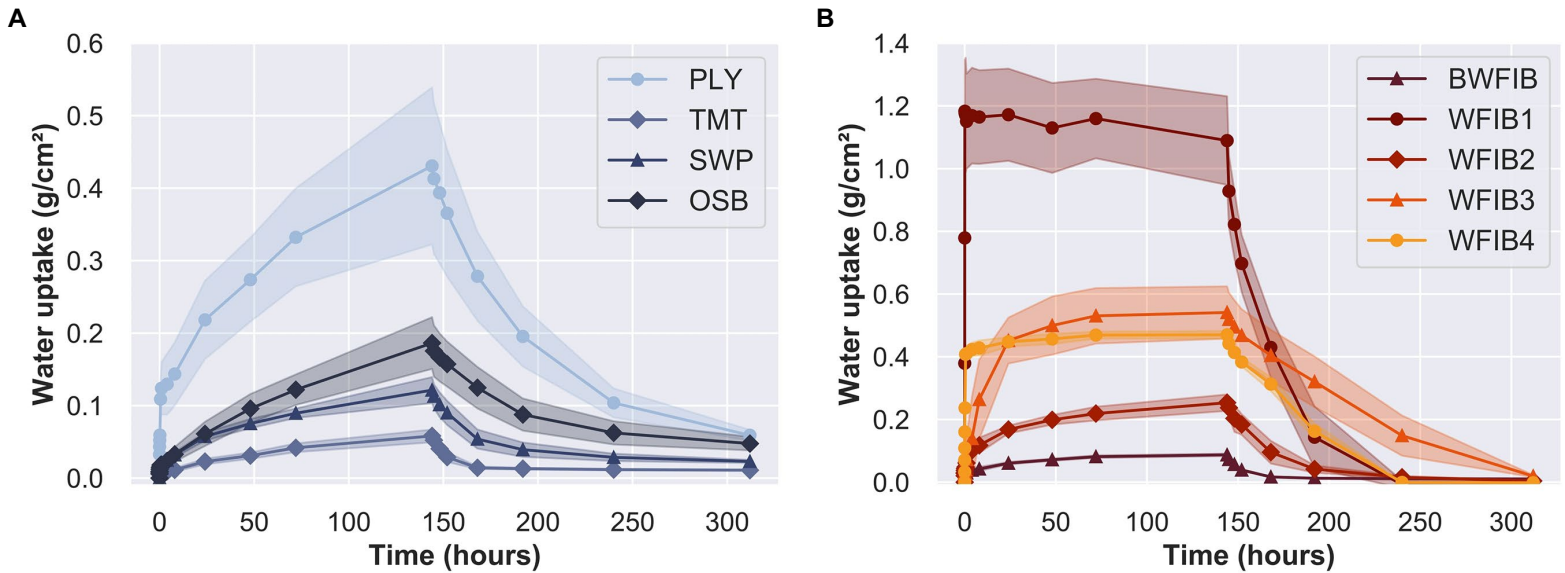
Mean liquid water uptake (g/cm^2^) with standard deviation over 144 h of absorption and 168 h of desorption in a floating test. **(A)** Wood-based panels: radiata pine plywood (PLY), thermally modified spruce (TMT), three-layer spruce panel (SWP) and oriented strand board (OSB); **(B)** Insulation materials: bituminised wood fibre board (BWFIB) and wood fibre insulation type 1–4 (WFIB1-4).

Water absorption and desorption properties were assessed with the floating test, during which the materials were laid afloat a water surface and the amount of absorbed water was registered at predetermined time intervals. The total amount of liquid water absorbed after 144 h differed substantially between wood-based materials, with some of the wood fibre insulation materials having a water uptake more than 20 times higher (Figure 8B) than those of the wood-based panels (Figure 8A). Solid wood with a lower density, and therefore a higher porosity, typically absorbs more water (Fredriksson, 2019). This principle applies to wood-based materials as well and was clearly observed for WFIB1, which had an estimated porosity of 97% (Table 2). WFIB2 and WFIB3 had similar overall porosities, but their water absorption and desorption properties were different. Clearly, other phenomena besides overall porosity affect the water absorption capacity of insulation materials and wood-based panels as well.

#### Solid Wood-Based Panels and Thermally Modified Wood

Radiata pine plywood (PLY) absorbed significantly more water during the floating test than the other wood-based panels and had a water uptake of 0.44 g/cm^2^ after 144 h of water absorption (Figure 8A). Parameter a_1_ in Eq. (3), affecting the steepness of the absorption curve, was more than four times higher (Table 4). A previous study by De Windt et al. (2018) showed that the moisture dynamics of plywood are highly dependent on the wood species of the (top) veneers. The water uptake of radiata pine plywood (0.43 g/cm^2^) indeed differed from the water uptake of birch plywood (0.24 g/cm^2^) and okoumé plywood (0.19 g/cm^2^) after 144 h of absorption, and was lower than that of solid radiata pine (0.67 g/cm^2^), as described by Van Acker et al. (2014).

**Table 4:** Absorption class, water uptake (g/cm^2^) at 144 h of absorption and parameters of fitted curves for absorption and desorption phases.

Label	Absorption Eq. (3)				Desorption Eq. (4)		
	Absorption class	144 h g/cm^2^	a_1_ (10^−5^ g/cm^2^)	b_1_ (−)	a_2_ (10^−5^ g/cm^2^)	b_2_ (10^−5^ g/cm^2^)	c_2_ (−)
PLY	7	0.43	9	0.30	5	38	51
TMT	1	0.06	1	0.46	1	5	8
SWP	4	0.12	2	0.41	3	9	21
OSB	6	0.19	1	0.57	5	14	42
BWFIB	2	0.09	3	0.20	1	7	8
WFIB1	8	1.09	97	0.06	−2	103	26
WFIB2	1	0.25	7	0.26	1	24	24
WFIB3	8	0.54	12	0.34	−28	80	162
WFIB4	7	0.47	30	0.12	−5	51	54

PLY, Plywood radiata pine; TMT, Thermally modified spruce; SWP, Three-layer spruce panel; OSB, Oriented strand board; BWFIB, Bituminised wood fibre board; WFIB1-5, Wood fibre insulation type 1–5.

The water uptake of the three-layer spruce panel (SWP) was slightly lower (0.12 g/cm^2^) than the values for solid Norway spruce (0.14 and 0.15 g/cm^2^) reported by Van Acker et al. (2014). SWP is composed of three solid wood boards glued together, while OSB is made of wood strands. These differences in material structure did not affect the initial water absorption phase, as the absorption curves of OSB and three-layer spruce panel were similar during the first 24 h of absorption. After 24 h, however, the OSB specimens absorbed water at a higher absorption rate. Its material structure (Figure 3B) and the presence of pores substantially larger than tracheids (Figure 3A) clearly affected its water absorption rate (Figure 8A). The water had desorbed faster in PLY than OSB, as indicated by the higher b_2_ (Table 4). PLY and OSB had a higher residual moisture (*ω*_r_) after 96 and 144 h of desorption (Table 5). Clearly, water is less easily released from OSB and plywood due to the material structure, which enhances the risk of water entrapment in practice (De Windt et al., 2018).

**Table 5:** Overview of the residual moisture (*ω*_r_; %) after 48, 96 and 144 h of desorption.

Label	*ω*_r,48_		*ω*_r,96_		*ω*_r,144_	
	Mean	Std	Mean	Std	Mean	Std
PLY	20.98	4.37	11.12	2.05	6.34	0.67
TMT	2.25	0.20	2.03	0.14	1.89	0.12
SWP	4.68	1.34	3.40	0.71	2.73	0.35
OSB	8.05	2.23	5.73	1.62	4.37	0.96
BWFIB	2.07	0.13	1.81	0.11	1.70	0.06
WFIB1	203.93	137.23	−11.65^*^	19.31	−13.06^*^	19.64
WFIB2	2.20	0.62	0.83	0.23	0.23	0.08
WFIB3	20.64	4.91	9.52	3.93	1.25	0.20
WFIB4	109.99	11.37	−0.51^*^	0.66	−1.26^*^	0.36
WFIB5	125.16	64.19	91.51	52.41	43.17	23.89

PLY, Plywood radiata pine; TMT, Thermally modified spruce; SWP, Three-layer spruce panel; OSB, Oriented strand board; BWFIB, Bituminised wood fibre board; WFIB1-5, Wood fibre insulation board type 1–5.

* Negative values indicate a possible mass loss due to leaching and fibre loss.

Thermally modified spruce (TMT) had the lowest water uptake after 144 h of absorption of all tested materials. Clearly, its increased pore surface hydrophobicity due to the thermal treatment decreased the water absorption, at least for the duration of the absorption phase of the floating test. During the desorption phase, most of the absorbed water in TMT had desorbed in the first 24 h of desorption, after which the desorption rate reduced. The water uptake after 144 h of absorption was similar as found in previous studies on thermally modified spruce (Van Acker et al., 2014) and corresponds to the general knowledge that thermal modification reduces the hygroscopicity of wood (Kekkonen et al., 2014; Hill et al., 2021). Note that outdoor exposure might affect the moisture performance of thermally modified wood, as was previously shown in a study by Žlahtič-Zupanc et al. (2018), in which the moisture content after 1 h of immersion was six times higher for weathered thermally modified spruce as compared to non-weathered thermally modified spruce.

#### Wood Fibre Insulation Materials

Due to its high porosity (Table 2) and its large pores (Figure 5), WFIB1 absorbed water fast and reached its full absorption potential after 5 min (Figure 8B). Since WFIB1 is made from loosely connected wood fibres and has a very low density (Table 2), the material collapsed during water absorption. The other wood fibre insulation materials were denser (Table 2) and had a more rigid structure, though clear signs of shrinking and swelling was observed during the experiment. For instance, more than 100% of the pore volume of WFIB4 was filled with water during the first hour of absorption, indicating that the material had swollen and could therefore contain more water than the calculated pore volume in dry state. Unlike the water uptake of WFIB1, which reached a plateau after 5 min, the water uptake of WFIB4 continued to increase up to 72 h of absorption after which it remained stable (Figure 8B). The LFNMR relaxation time distributions indicated that water occupied smaller pores in WFIB4 as compared to WFIB1 in water-saturated state, possibly explaining the presence of this secondary absorption phase after the primary absorption phase. Also, WFIB4 was 14% less porous than WFIB1 in dry state (Table 2). The lower water uptake is likely due to the smaller thickness of WFIB4 as compared to WFIB1. Indeed, when normalized over sample thickness, the moisture concentration of WFIB4 is two times higher than WFIB1 (Supplementary Figure 3).

The water uptake of WFIB2 and BWFIB was more than four times lower than that of WFIB1, even though WFIB2 and BWFIB had similar LFNMR relaxation time distributions as WFIB1. Likewise, the water uptake of WFIB3 was twice as low as WFIB1, even though it contained the largest pores in water-saturated state. This large difference in water absorption rate is likely related to the hydrophobic properties and manufacturing process of each material. Indeed, WFIB3 and BWFIB contain paraffin and bitumen, respectively. The bitumen treatment appeared to be more effective as a water repellent than the paraffin treatment for these specific materials. However, as WFIB2 does not contain any additives, the manufacturing process itself also played an important role. WFIB1 is a loose wood fibre mat with a density of 50 kg/m^3^, in which water can enter easily and the fibres stick together. In contrast, WFIB2 is a dense material, with the fibres organised in layers, as can be seen in Figure 5B. Presumably, these layers or the fibre bonds in general prevent the water from penetrating deeply into the material. It seems that the wet manufacturing process prevented water uptake more than the dry manufacturing process with isocyanate in combination with paraffin as additive (WFIB3), possibly due to a better fibre-fibre binding.

**Figure 9:**
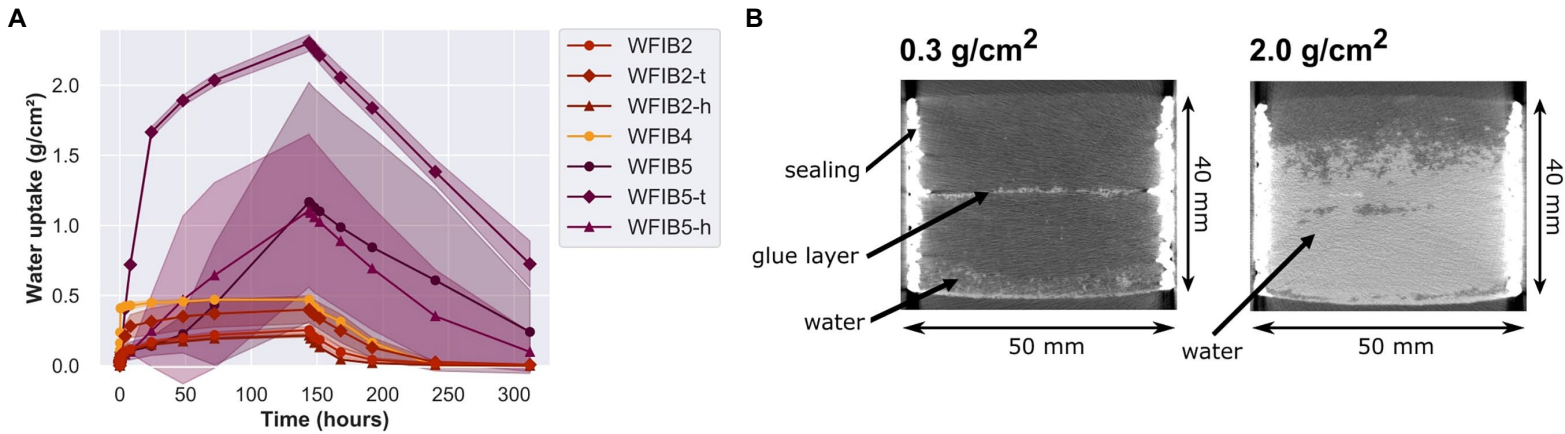
**(A)** Mean liquid water uptake (g/cm^2^) with standard deviation over 144 h of absorption and 168 h of desorption in a floating test. Comparison of wood fibre insulation boards made using the wet manufacturing process (WFIB2, WFIB4 and WFIB5). Specimens were laid atop the water surface with the densified bottom side, without (−) or with a hole (h) or with the undensified top (t) in contact with the water surface; **(B)** Difference in water absorption between a wood fibre insulation board type 5 (WFIB5) specimen which had absorbed 0.3 g/cm^2^ and a specimen which had absorbed 2.0 g/cm^2^ after 144 h of absorption. X-ray CT scan taken after 144 h of absorption and 1 h of desorption.

The wet manufacturing process causes a densification of the bottom fibres, which might also affect the water uptake. We therefore assessed whether the lower water uptake was caused by better fibre-fibre binding or whether it was related to the densified bottom layer. Figure 9A illustrates the liquid water uptake (g/cm^2^) of three WFIBs manufactured with a wet manufacturing process. Clearly, the wet manufacturing process does not necessarily result in a stronger fibre-fibre binding, as there was a high variability in the moisture dynamics of WFIB5, with values going from 0.5 to 2.4 g/cm^2^ (Figures 9A,B). When the softer top of the WFIB2 specimens was put in contact with water instead of the densified bottom, the mean water absorption after 144 h increased from 0.25 to 0.4 g/cm^2^. However, when the softer top of the WFIB5 specimens was put in contact with water, water absorptions of up to 2.4 g/cm^2^ occurred. Interestingly, when exposed to the softer side, the variability was limited. Consequently, the variability of WFIB5 in water contact with the hard bottom side, was likely due to inconsistencies in the harder bottom layer. Note that presence of bore holes did not affect the water absorption significantly, as the water absorption was within the error margin of the specimens without a bore hole, both for WFIB2 and WFIB5. As the water absorption of the WFIB2 specimens with the top side in water contact was still below 0.5 g/cm^2^, the main influence of the wet manufacturing process was not related to the thicker bottom layer, but to better fibre-fibre binding throughout the specimen. Presumably, the addition of paraffin in WFIB5 had a negative impact on the bonding strength between fibres in the wet manufacturing process, making it easier for water to enter, although it could also be that the wet manufacturing process in general provides inconsistent water uptake results. Note that the low water uptake of WFIB4 is misleading, as the WFIB4 specimens were water saturated and could not take up more water due to the limited panel thickness (5 mm).

### Implications for Service Life

In an extensive study on the moisture dynamics of plywood, a strong correlation was found between the results from the floating test and the actual moisture dynamics of plywood in outdoor exposure (De Windt et al., 2018). Based on residual moisture (*ω*_r_), use class recommendations (CEN EN 335 standard, 2013) were given related to the moisture dynamics of plywood. For instance, plywood with an *ω*_r_ lower than 3.5% was suggested to be suited for extended service life in UC3.1 and UC3.2 (outdoor exposure in limited and persistent moisture conditions, respectively), while a *ω*_r_ between 3.5 and 5.5% would be suited for a short to average service life in UC3.1. In addition to *ω*_r_, other parameters could be used to predict service life as well. The absorption class, derived from the water uptake after 144 h of absorption, gives an indication of the water absorption capacity of a material (Table 4). Furthermore, several absorption and desorption parameters were derived to better describe the moisture dynamics of the assessed materials.

TMT, BWFIB and WFIB2 performed very well on the moisture dynamics indicators. TMT and WFIB2 were classified in absorption class 1, and BWFIB in absorption class 2, indicating a low amount of water uptake during the absorption phase (Table 4). These values corresponded to those found by Van Acker et al. (2014) for ipé, teak, walaba and thermally modified spruce, poplar and pine wood. Additionally, TMT and BWFIB had low c-values for desorption, indicating that they are fast-drying materials. The *ω*_r_ after 48 h for the three materials was below 2.5%. De Windt et al. (2018) classified plywood with an *ω*_r_ below 2.5% after 72 h of desorption, as a material that is expected to have a long service life in UC1 (indoor applications) to UC3 (outdoor exposure), and even UC4. Since the durability of BWFIB and WFIB2 against Basidiomycetes is low (De Ligne, 2021), it should not be recommended in water or ground contact (UC4). Assuming that the same criteria are applicable to TMT, BWFIB, and WFIB2, our results indicate that these particular materials can be used up to UC3.2 (above ground, exposed to prolonged wetting conditions). Indeed, TMT is often applied as cladding in outdoor exposure conditions (Jones et al., 2018) and BWFIB as roof insulation boards. Nevertheless, the structural integrity and insulating capacity of wood fibre insulation materials might become affected due to subsequent wetting and drying. Though the wood fibre insulation boards did not disintegrate during the floating test, the pore distribution and fibre connections were likely affected, possibly impacting the insulating capacity.

WFIB1, WFIB3, and WFIB4 absorbed much water and were classified in absorption classes 7 and 8 (Table 4). Although the water uptake of WFIB1 was twice as high as of WFIB3, both were classified in absorption class 8. It could be recommended to expand the absorption classes to better differentiate water absorption in wood-based insulation materials. Nonetheless, all wood fibre insulation materials were able to dry to an *ω*_r_ below 2.5% after 144 h of desorption, with the exception of WFIB5 (Table 5). WFIB1 and WFIB4 already reached an *ω*_r_ below 2.5% after 96 h of desorption. UC2 (indoor applications with a condensation risk) and possibly UC3.1 (outdoor exposure, limited moisture) could be recommended, on the condition that the insulating capacity is not affected due to subsequent wetting and drying. The absorption class of PLY, OSB, and SWP was 7, 6 and 4, respectively. While SWP reached an *ω*_r_ below 3.5% after 96 h and below 2.5% after 144 h, the *ω*_r_ in PLY remained higher than 5.5% after 144 h. Water accumulation might be an issue for these specific OSB and PLY products.

### Suitability of the Applied Techniques

LFNMR provided useful insights into the pore distribution of nine commonly used wood-based building materials in water-saturated state, including cell wall water. Since LFNMR relaxation time distributions give an indication of both pore size and the affinity of the water molecules with the pore surface, it is often difficult to interpret the results. For future experiments, more controlled testing of specific phenomena of interest are recommended, to determine, for instance, the influence of changing one step in the manufacturing process or by comparing the LFNMR relaxation time distributions of experimentally designed test materials where all components have remained the same, except for pore size.

X-ray CT was very useful to visualize the internal pore space of the materials in dry state and proved to be valuable to confirm pore distributions of wood-based panels and thermally modified wood. However, for wood fibre insulation materials, the proposed X-ray CT method was not suited to gain insight into the pore volume in water-saturated state, as fibres stick together and the pore volume changes when wet. Therefore, the X-ray CT pore size distributions of the wood fibre insulation materials could not be compared with the LFNMR data. For future work, it is recommended to use X-ray CT to assess the pore space of the dry material before and after water saturation. Nevertheless, for loose, fibrous materials, LFNMR might be the only method to quantify pore distributions in water-saturated state, though MRI visualization and future advancements in development of contrast fluids for X-ray CT, spectral and dark-field X-ray CT might be a solution as well.

ATR-FTIR is a useful technique to assess the impact of the manufacturing process on the composition of a wood-based material. It can also provide information on whether or not additives are homogenously present in the material. ATR-FTIR measurements would be most beneficial to assess the effect of a change in the manufacturing process, the amount or type of additives or the degree of modification.

While LFNMR, X-ray CT and ATR-FTIR are well-suited to gain fundamental insights in material moisture dynamics, the floating test is a relevant test for comparison of liquid water absorption and subsequent desorption of wood-based building materials as an indication of material in contact with liquid water in service (rain, leakages, condensation). In order to use it for actual service life prediction of different types of wood-based materials, floating test results would need to be related to actual exposure conditions and service life in practice. Furthermore, most wood-fibre insulation materials are applied in dry conditions (UC1), in conditions with a condensation risk (UC2) or in conditions with a risk of leakages and rain water infiltration, such as roof insulation (up to UC3). The floating test is suited for the latter applications, but when it comes to condensation in wood-based insulation materials (UC2), a different type of test might be better suited. Also, the floating test is not appropriate for materials that lose their consistency in liquid water contact, such as loose wood fibre or cellulose fibre insulation. When a new test specifically assessing condensation in wood-fibre insulation materials is developed, it would ideally also take structural integrity and insulation capacity into account.

## Conclusion

The assessed commercially available wood-based materials showed a wide range of moisture dynamics, proving the potential for tailoring wood-based materials towards fit-for-purpose moisture performance. Water absorption and desorption characteristics of wood-based materials were not only dependent on overall porosity but were influenced by the materials’ hydrophobic properties, manufacturing process and pore size distribution. Thermal modification and hydrophobic additives had a major impact on the water absorption and desorption characteristics (TMT, WFIB3 and BWFIB). We were able to prove that when the wood anatomy of a wood species was not altered substantially when producing the wood-based panel product, the water populations of the wood-based panel in water-saturated state were similar to those of solid wood, as was the case for plywood and the three-layer spruce panel. However, the material structure of plywood had a clear impact as it reduced water uptake during absorption but contributed to water entrapment in the desorption phase as water was released slowly. In contrast, all but one wood fibre insulation material had excellent desorption properties due to their high porosity. Service life predictions were given based on the residual moisture criteria described in De Windt et al. (2018). Assuming that these are applicable to wood-based building materials in general, TMT, BWFIB and WFIB2 would be expected to have an extended service life up to UC3.2 (outdoor exposure in moist conditions). For WFIB1 and WFIB4 UC2 (interior, possibility of condensation) and possibly UC3.1 (outdoor exposure, limited moist conditions) would be recommendable. However, to guarantee an extended service life, the moisture dynamics as assessed by the floating test should be compared to actual moisture performance in outdoor exposure conditions for these materials, or at least for a selection of reference materials.

## Data Availability Statement

The raw data supporting the conclusions of this article will be made available by the authors, without undue reservation.

## Author Contributions

LL, JVB, JA, ET, and LT: conceptualization. LL, JMB, JVB, BB, JA, ET, and LT: methodology. LL and LT: software. LL: validation, formal analysis, data curation, writing—original draft preparation, visualization, and project administration. LL and SO: investigation. JVB, JA, ET, and LT: resources. JMB, JVB, BB, JA, SO, ET, and LT: writing—review and editing. JMB, JVB, BB, JA, ET, and LT: supervision. LL, JMB, JVB, BB, and JA: funding acquisition. All authors have read and agreed to the published version of the manuscript.

## Funding

This research was funded by Research Foundation Flanders (FWO SB grant number 1S53417N) and BOF Special Research Fund for RL (BOF Starting Grant JVdB, BOFSTG2018000701). The Special Research Fund of Ghent University is acknowledged for the support to the UGCT Centre of Expertise (BOF.EXP.2017.0007).

## Conflict of Interest

The authors declare that the research was conducted in the absence of any commercial or financial relationships that could be construed as a potential conflict of interest.

## Publisher’s Note

All claims expressed in this article are solely those of the authors and do not necessarily represent those of their affiliated organizations, or those of the publisher, the editors and the reviewers. Any product that may be evaluated in this article, or claim that may be made by its manufacturer, is not guaranteed or endorsed by the publisher.

## Footnotes

^1^ http://www.ugct.ugent.be

^2^ https://www.python.org/
