# Supplementary material for: Moisture Dynamics of Wood-Based Panels and Wood Fibre Insulation Materials
Source: Front Plant Sci. 2022 Jul 14;13:951175. doi: 10.3389/fpls.2022.951175 (PMC9330446; doi:10.3389/fpls.2022.951175)
Supplement: Supplementary file 1 [file Data_Sheet_1.pdf]

# *Supplementary Material*

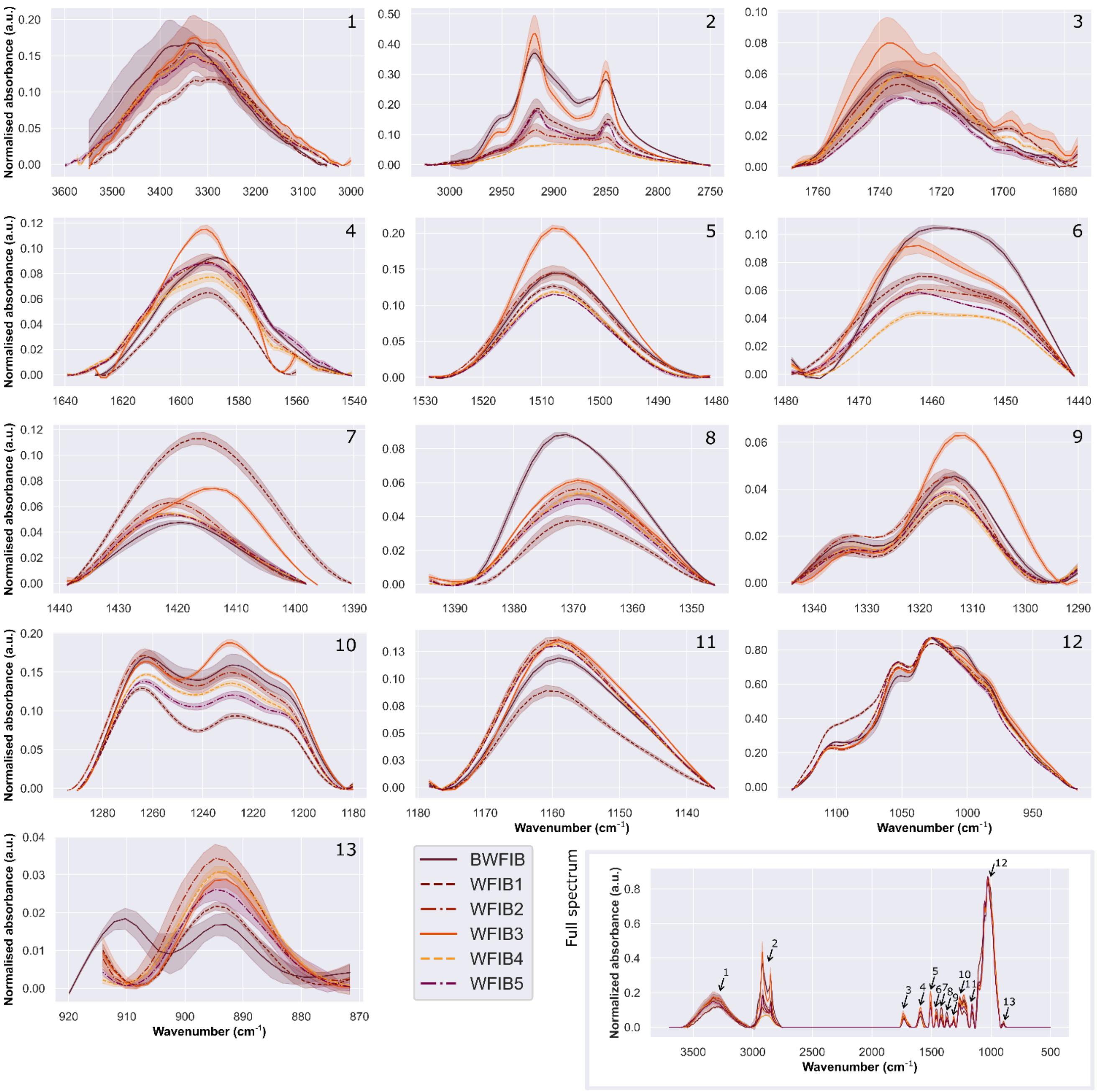


**Supplementary Figure 1.** Baseline-corrected, rescaled, smoothed, mean FTIR spectrum with standard deviation of wood fibre insulation boards (BWFIB and WFIB1-5), with 13 zoomed-in regions of interest.

**Expand and shrink approach**

First, all white voxels located next to a black voxel expand n times with 1 voxel. Next, all white voxels located next to a black voxel shrink n times with 1 voxel in Octopus analysis. Simply put, if expanded one time (n=1, see Figure 2 in 2-D), small pores (black voxels) are removed by the surrounding white voxels, while larger pores became smaller. For each n consecutive expanding and n consecutive shrinking steps, the total pore volume was assessed. The number of consecutive expanding and shrinksing steps (n) was increased until no more pores remained (pore volume of 0.02%).

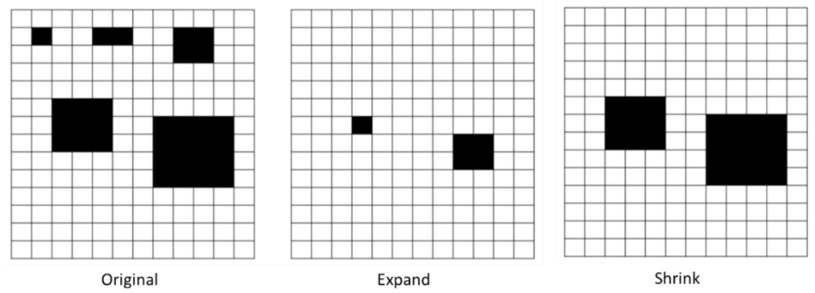


**Supplementary Figure 2.** Illustration expand and shrink approach with n=1.

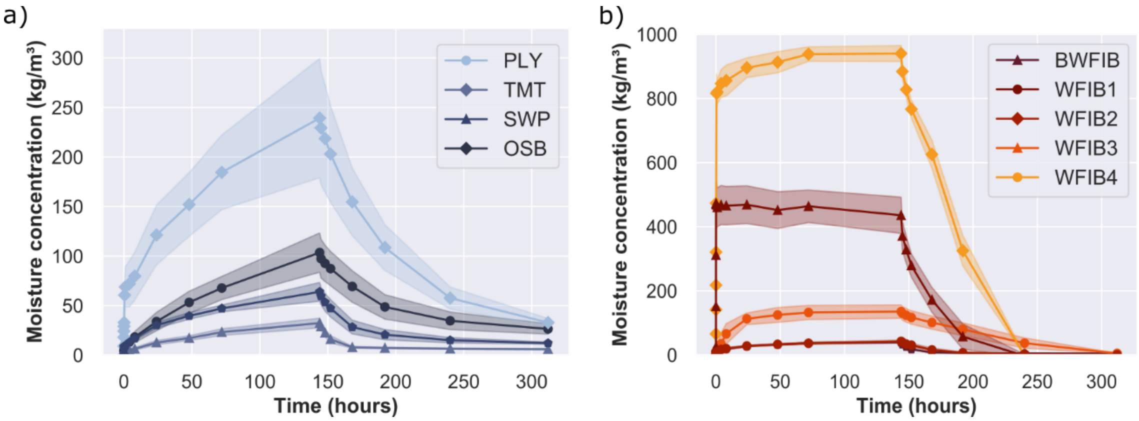


**Supplementary Figure 3.** Mean moisture concentration (kg/cm³) with standard deviation over 144 hours of absorption and 168 hours of desorption in a floating test. a) Wood-based panels: radiata pine plywood (PLY), thermally modified spruce (TMT), three-layer spruce panel (SWP) and oriented strand board (OSB). b) Insulation materials: porous bituminised wood fibre board (BWFIB) and wood fibre insulation type 1-4 (WFIB1-4).

**Supplementary Table 1.** Mean values of $T_2$ (ms) determined by continuous curve fitting and reference values for solid radiata pine and Norway spruce wood. The Kruskal-Wallis H-test was applied for testing differences in median $T_2$ values for each peak, with Dunn's multiple comparison test as post-hoc test with Benjamini-Hochberg correction. Based on Dunn's multiple comparison test, the materials were grouped (a-h). For each material, the assigned group letter is put in bold, while the other letters indicate the materials which had similar (= not significantly different) $T_2$ values. LW = latewood, EW = earlywood.

| | **Peak 1** | | **Peak 2** | | **Peak 3** | | **Peak 4** | | **Peak 5** | | **Peak 6** | |
|---|---|---|---|---|---|---|---|---|---|---|---|---|
| | **mean** | **std** | **mean** | **std** | **mean** | **std** | **mean** | **std** | **mean** | **std** | **mean** | **std** |
| **PLY** | 3.4 $^{\mathbf{a},b,c}$ | 0.1 | 30 $^{\mathbf{a},b}$ | 3.6 | 101 $^{\mathbf{a},b,c}$ | 7.8 | | | | | | |
| **TMT** | 1.3 $^{b,c,\mathbf{d},e}$ | 0.3 | 26 $^{\mathbf{a},b}$ | 8.9 | 97 $^{\mathbf{a},b,c}$ | 12.8 | | | | | | |
| **SWP** | 0.8 $^{c,d,\mathbf{e}}$ | 0.1 | 7 $^{b,\mathbf{c}}$ | 3.4 | 57 $^{a,b,\mathbf{c},d,e}$ | 20.0 | | | | | | |
| **OSB** | 1.8 $^{a,\mathbf{b},c,d}$ | 0.2 | 11 $^{a,\mathbf{b},c}$ | 2.0 | 69 $^{a,\mathbf{b},c,d}$ | 7.1 | 263 $^{\mathbf{a},b}$ | 59.0 | | | | |
| **BWFIB** | 1.1 $^{b,c,\mathbf{d},e}$ | 0.5 | 85 $^{b,\mathbf{c}}$ | 4.0 | 38 $^{b,c,\mathbf{d},e}$ | 12.4 | 123 $^{b,\mathbf{c}}$ | 41.1 | 482 $^{a,\mathbf{b},c}$ | 45.4 | | |
| **WFIB1** | 1.8 $^{a,b,\mathbf{c},d,e}$ | 0.6 | 10 $^{a,\mathbf{b},c}$ | 5.1 | 46 $^{b,c,\mathbf{d},e}$ | 10.5 | 200 $^{a,\mathbf{b},c}$ | 81.5 | 728 $^{\mathbf{a},b}$ | 141.3 | | |
| **WFIB2** | 1.1 $^{b,c,\mathbf{d},e}$ | 0.6 | 5 $^{b,\mathbf{c}}$ | 3.1 | 32 $^{c,d,\mathbf{e}}$ | 11.0 | 119 $^{a,\mathbf{b},c}$ | 16.9 | 398 $^{b,\mathbf{c},d}$ | 61.1 | | |
| **WFIB3** | 1.4 $^{a,b,\mathbf{c},d,e}$ | 0.3 | 7 $^{b,\mathbf{c}}$ | 2.5 | 35 $^{c,d,\mathbf{e}}$ | 10.0 | 93 $^{b,\mathbf{c}}$ | 16.1 | 282 $^{c,\mathbf{d}}$ | 41.2 | 937 | 46.9 |
| **WFIB4** | 1.8 $^{a,b,\mathbf{c},d,e}$ | 0.9 | 13 $^{a,\mathbf{b},c}$ | 8.4 | 70 $^{a,\mathbf{b},c,d}$ | 11.6 | 287 $^{\mathbf{a},b}$ | 59.3 | | | | |
| **Reference** | | | | | | | | | | | | |
| **Radiata pine[1]** | 3 | | 20-30 | | 100-110 | | | | | | | |
| **Norway spruce[2]** | 1.3-2.2 | | 4.8-18.2 | | LW: 45.4-77.4<br>EW: 57.6-103.8 | | | | | | | |

[1]Beck et al (2018); [2]Fredriksson and Thygesen (2017)

**Supplementary Table 2.** Mean relative sum of amplitudes (-) under each peak determined by continuous curve fitting. The Kruskal-Wallis H-test was applied for testing differences in median relative area for each peak, with Dunn's test as post-hoc with Benjamini-Hochberg correction. Based on Dunn's multiple comparison test, the materials were grouped (a-h). For each material, the assigned group letter is put in bold, while the other letters indicate the materials which had similar (=not significantly different) areas.

| | **Peak 1** | | **Peak 2** | | **Peak 3** | | **Peak 4** | | **Peak 5** | | **Peak 6** | |
|---|---|---|---|---|---|---|---|---|---|---|---|---|
| | **mean** | **std** | **mean** | **std** | **mean** | **std** | **mean** | **std** | **mean** | **std** | **mean** | **std** |
| **PLY** | 0.16 $^{a,\mathbf{b},c,d}$ | 0.02 | 0.15 $^{\mathbf{a},b,c}$ | 0.02 | 0.69 $^{a,\mathbf{b},c,d,e}$ | 0.04 | | | | | | |
| **TMT** | 0.06 $^{b,c,\mathbf{d},e}$ | 0.01 | 0.11 $^{a,\mathbf{b},c,d}$ | 0.04 | 0.84 $^{\mathbf{a},b,c,d}$ | 0.04 | | | | | | |
| **SWP** | 0.16 $^{a,\mathbf{b},c,d}$ | 0.03 | 0.05 $^{a,b,\mathbf{c},d,e}$ | 0.02 | 0.77 $^{a,b,\mathbf{c},d,e,f}$ | 0.07 | | | | | | |
| **OSB** | 0.24 $^{\mathbf{a},b,c}$ | 0.05 | 0.05 $^{a,b,\mathbf{c},d,e}$ | 0.02 | 0.54 $^{a,b,c,\mathbf{d},e,f,g}$ | 0.10 | 0.12 $^{b,\mathbf{c},d}$ | 0.02 | | | | |
| **BWFIB** | 0.06 $^{b,c,\mathbf{d},e}$ | 0.02 | 0.03 $^{b,c,\mathbf{d},e}$ | 0.01 | 0.12 $^{d,e,f,\mathbf{g},h}$ | 0.06 | 0.23 $^{a,\mathbf{b},c}$ | 0.06 | 0.58 $^{\mathbf{a},b}$ | 0.06 | | |
| **WFIB1** | 0.05 $^{c,d,\mathbf{e}}$ | 0.01 | 0.03 $^{b,c,\mathbf{d},e}$ | 0.01 | 0.20 $^{b,c,d,\mathbf{e},f,g,h}$ | 0.07 | 0.13 $^{b,\mathbf{c},d}$ | 0.02 | 0.61 $^{\mathbf{a},b}$ | 0.02 | | |
| **WFIB2** | 0.04 $^{c,d,\mathbf{e}}$ | 0.02 | 0.04 $^{b,c,\mathbf{d},e}$ | 0.02 | 0.15 $^{c,d,e,\mathbf{f},g,h}$ | 0.04 | 0.27 $^{a,\mathbf{b},c}$ | 0.03 | 0.55 $^{a,\mathbf{b},c}$ | 0.10 | | |
| **WFIB3** | 0.06 $^{b,c,\mathbf{d},e}$ | 0.01 | 0.01 $^{c,d,\mathbf{e}}$ | 0.01 | 0.04 $^{e,f,g,\mathbf{h}}$ | 0.01 | 0.06 $^{c,\mathbf{d}}$ | 0.02 | 0.10 $^{b,\mathbf{c}}$ | 0.01 | 0.75 | 0.01 |
| **WFIB4** | 0.07 $^{a,b,\mathbf{c},d,e}$ | 0.02 | 0.10 $^{a,\mathbf{b},c,d}$ | 0.07 | 0.45 $^{a,b,c,\mathbf{d},e,f,g}$ | 0.06 | 0.38 $^{\mathbf{a},b}$ | 0.04 | | | | |
| **Reference** | | | | | | | | | | | | |
| **Radiata pine**[1] | 0.10-0.27 | | 0.01-0.04 | | 0.63-0.85 | | | | | | | |
| **Norway spruce**[2] | 0.16 $^{a,\mathbf{b},c,d}$ | 0.02 | 0.15 $^{\mathbf{a},b,c}$ | 0.02 | 0.69 $^{a,\mathbf{b},c,d,e}$ | | | | | | | |

[1]Beck et al (2018); [2]Fredriksson and Thygesen (2017)
